# Resist-Free Shadow Deposition Using Silicon Trenches for Josephson Junctions in Superconducting Qubits

**DOI:** 10.1021/acs.nanolett.6c02734

**Published:** 2026-10-22

**Authors:** Tathagata Banerjee, Stephen Daniel Funni, Saswata Roy, Judy J. Cha, Valla Fatemi

**Affiliations:** † School of Applied and Engineering Physics, 5922 Cornell University, Ithaca, New York 14853, United States; ‡ Department of Materials Science and Engineering, Cornell University, Ithaca, New York 14853, United States; § Department of Physics, Cornell University, Ithaca, New York 14853, United States

**Keywords:** Josephson junction, superconducting qubit, aluminum, nanofabrication

## Abstract

Superconducting qubit fabrication innovations continue to be explored to achieve higher performance. Despite improvements to base layer fabrication and processing, resist-based Josephson junction (JJ) schemes have largely remained unchanged. The polymer mask during deposition causes chemical contamination and limits *in situ* and *ex situ* surface preparation, junction materials, and scalability. Here, we demonstrate a resist-free approach to junction fabrication based on etched silicon trenches that is compatible with standard silicon microfabrication processes and easily integrated into existing innovations in qubit base layer fabrication and chemical processing. We fabricate Al-AlO_*x*_-Al JJs and qubits using this method, measuring median energy relaxation times up to 188 μs. We find minimal contamination at the substrate–metal interface and fluctuations of energy relaxation on a 45 h time scale that are narrow and normally distributed. The method widens the process window for substrate preparation and new materials platforms.

## Graphical abstract

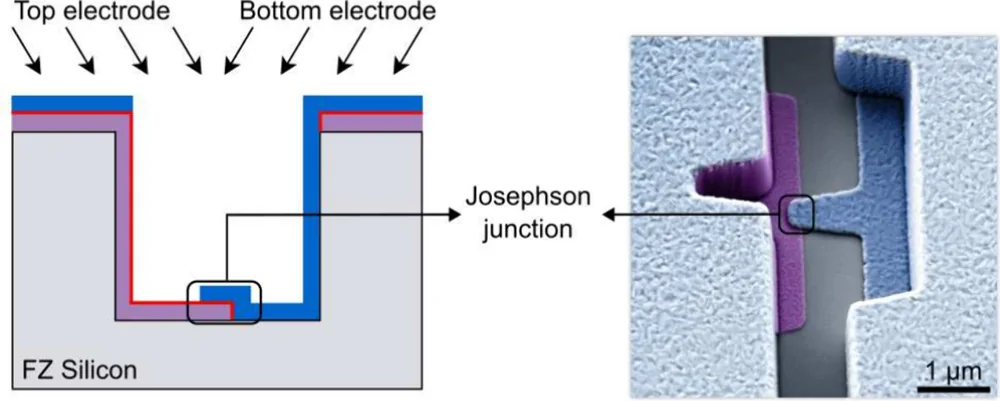

Solid state qubit platforms offer scalable approaches to quantum computation. Superconducting qubits in particular have demonstrated beyond break-even scaling of quantum error correction.^1,2^ For the qubit array demonstrations of quantum error correction, a majority of the errors that limit performance of the error correction code arise due to the coherence times not being long enough compared to gate operation times.^2^ Therefore, it remains imperative to continue improving performance at the single qubit level.

The past decade has seen substantial improvements to superconducting qubit coherence through innovations in materials choices and nanofabrication processing.^3–8^ In transmon qubits, these efforts have so far mainly targeted microwave losses originating at the surfaces of the “base layer” of the superconductor that account for the majority of the capacitance of the qubit. This has involved a multifaceted exploration on the choice of the base layer metallization, deposition methods, etching processes, and postfabrication cleaning.^6–11^ The investigations have so far culminated in transmon qubits with quality factors averaging 15 million and energy relaxation times at the millisecond scale,^3,8^ substantially surpassing the single-qubit coherence scale of scaled processors.^2^ In these qubits, *in operando*, the relaxation times fluctuate over time by factors of 3 or more relative to the mean in a non-Gaussian distribution.^4,7,12–14^ These fluctuations will limit the ability of superconducting qubits to scale up further as they will dominate errors and impose frequent recalibration duty cycles.

In contrast to the base layers, the fabrication process for the Josephson junction (JJ) has remained largely unchanged over the last 25 years. Indeed, the materials used—aluminum (Al) metal electrodes and the native surface aluminum oxide for the tunnel barrier—have been identical since the first superconducting qubit.^15^ This polymer liftoff-based fabrication process involves a double-angle deposition with an intermediate oxidation step. The principal advantage of this process is its simplicity, with a single lithography step and minimal number of process parameters, which makes process optimization relatively straightforward. However, the presence of the polymer mask results in chemical contamination and severely limits options for surface preparation, junction materials, deposition methods, and other fabrication process windows. Bridge-free resist-based bilayers have also been explored for JJ evaporation and show comparable performance to the Dolan-bridge method, but the limitations due to the presence of the resist persist.^16,17^ Other fully subtractive methods, such as the trilayer process, have been explored,^18–21^ but so far they are hindered by the substantially larger number of process steps which must be optimized to achieve sufficiently high quality factor devices to be competitive.

**Figure 1:**
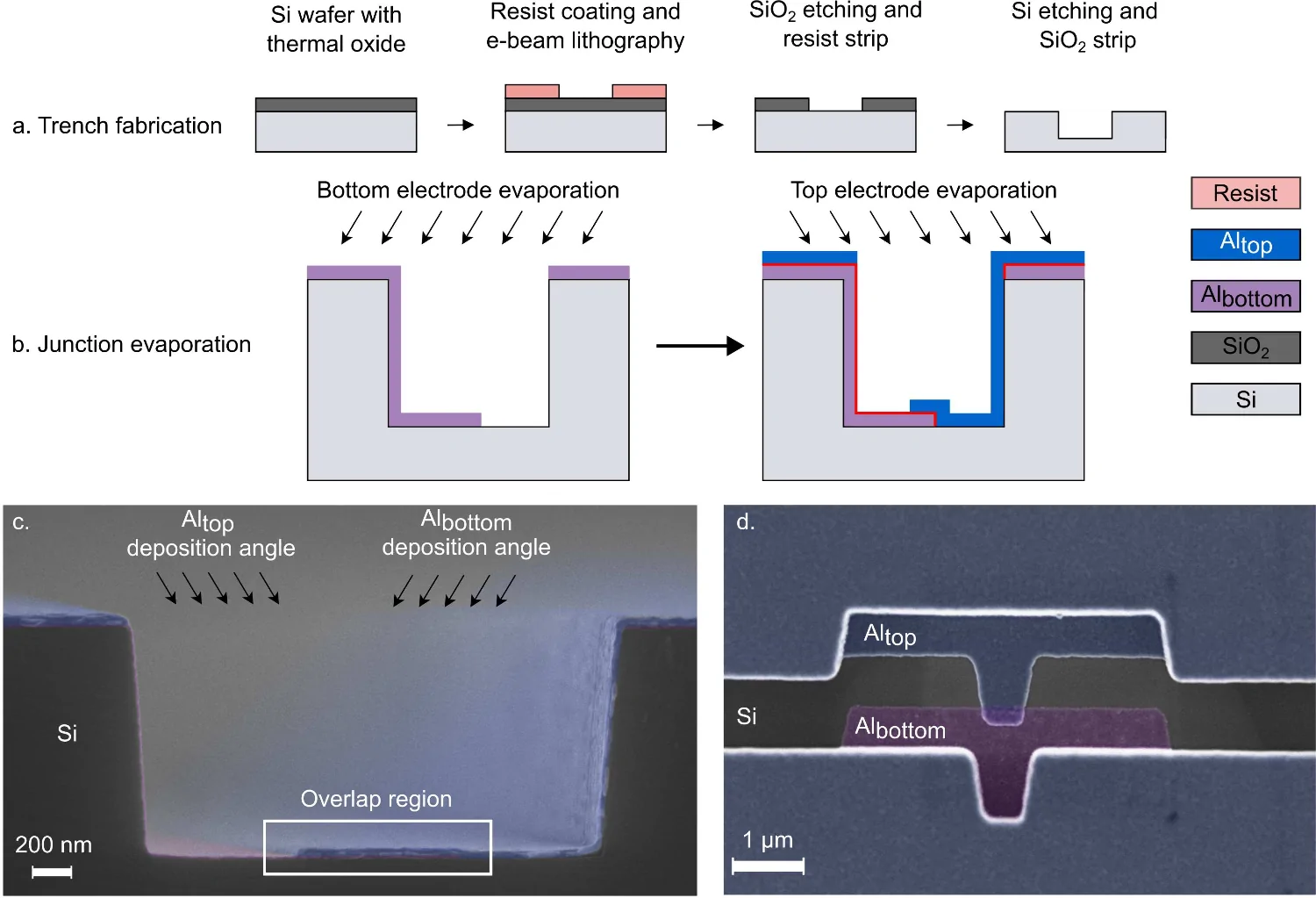
Josephson junction fabrication process. (a) Trench fabrication: a silicon wafer is thermally oxidized to form SiO_2_, followed by e-beam lithography and etching to form the hard mask. The Si is dry-etched followed by removal of the hard mask using hydrofluoric acid. (b) Trench-based junction evaporation: Al is evaporated at two angles to create an overlap at the bottom of the trench which forms the junction. (c) Cross-sectional false-colored electron micrograph of the JJ formed within the silicon trench. (d) Top-down false-colored electron micrograph of the JJ. When the trench narrows, the shadowed region extends beyond the midpoint of the trench, leading to no overlap. Asymmetrical electrode shapes are designed to ensure minimal effect due to any rotational misalignment during loading and deposition.

The limitations of both the liftoff and subtractive processes in use today provides an opportunity for an approach that fulfills the need for broader process exploration and optimization for junction fabrication while maintaining the simplicity of single-step shadow-evaporation. Ideally, such a process would be relatively straightforward, involve only a few nanofabrication steps, be compatible with now-standard angle evaporation tools, and take advantage of a fully inorganic, process resilient environment. There have also been efforts with inorganic stencil masks which show promise,^22–24^ but remain highly complicated fabrication processes, and may have mechanical challenges and large mask-substrate distances.^23^ So far, a simple, nanometrically precise approach that demonstrates leading-edge performance has remained elusive.

Here, we demonstrate a new, relatively simple approach to accomplishing single-step Josephson junction formation with only a trenched silicon substrate to enable the shadowing effect. The preparation of the substrate trench uses standard, clean complementary metal-oxide-semiconductor (CMOS) methods. The JJ deposition can then occur after surface preparation to ensure full removal of the surface oxide. We fabricate Al-AlO_*x*_-Al JJs with no detectable carbon or oxygen at the metal-substrate interface. Finally, we conduct a subtractive-only process to define transmon qubits with quality factors as high as 3 million (*T*_1_ averaging 188 μs). Our novel, clean, simple nanofabrication approach opens a broad landscape of processing conditions, surface preparation, and materials choices.

The junction fabrication scheme is based on etching trenches into the Si substrate. The sidewalls of the trench provide the necessary shadowed area for an overlap region to form the junction while leaving nonoverlapped regions for the two electrodes to climb the sidewall onto the top of the substrate. The formation of the overlap is controlled by modulating the width of the trench. When the trench narrows, the shadowed region extends beyond the midpoint of the trench, at which point no overlap is formed. Thus, we can define the width of the junction changing by the width of the trench, and the length of the junction by the length of the wider trench before it narrows.

The chip is first prepared by etching the trench into the substrate, as shown in Figure 1(a). We thermally oxidize a 4-in. high-resistivity Si(100) wafer to grow SiO_2_ for a hard mask. We define an etch mask with an electron-beam lithography process, followed by a CHF_3_/O_2_-based dry-etch process to transfer the pattern onto the hard mask. The Si is then etched with the SiO_2_ hard-mask using an HBr/Ar etch designed to maintain a smooth silicon surface. The trench formed is 1.2 μm deep, with the width varying between 1 μm away from the JJ (where there is no overlap) to 2.75 μm where the JJ overlap is formed. The sidewall angle is approximately 94°–95° at the top and bottom corners. Finally, the SiO_2_ is stripped using 10:1 hydrofluoric acid (HF). We note that many variants of silicon etch could be used to accomplish this step, with all steps being fully compatible with standard silicon microfabrication processes. The trench etching and fabrication is done on a wafer-scale, followed by dicing into individual chips for JJ evaporation and qubit fabrication. See Supporting Information 1 for further details of the trench etching process.

Immediately prior to junction formation, the chip is soaked in 10:1 buffered oxide etch (BOE) to strip any native SiO_2_ and remaining resist residue to ensure a clean substrate. The 20 nm (80 nm) aluminum for the bottom (top) electrode is evaporated at 49° (−49°) at a rate of 2 Å/s [Figure 1(b)]. Between depositions, the bottom electrode is oxidized at 1 Torr for 5 min using 100% O_2_. Figure 1(c) shows a cross-sectional SEM image of a 3 μm wide trench with the evaporated layers and a clear overlap region.

To form the JJ without any additional subtractive processing, the trench has a variable width. This is shown in Figure 1(d) with a top-down SEM image of a JJ formed by the process. The overlap region is formed at the widest part of the trench, and narrower parts of the trench result in only single layers of aluminum elsewhere in the trench or on the sidewalls. The asymmetric electrode shapes are chosen to ensure that small rotational misalignment of the chip during deposition produces minor changes to the junction shapes. We also remark that we do not observe the “halo” of hydrocarbons often seen when using resist-based liftoff.^25^

Room temperature two-probe measurements are conducted on junctions of varying areas and oxidation doses (see Supporting Information 2). The JJ shown in Figure 1(d), with dimensions of 360 nm × 180 nm and the above dose, has a resistance of around 6.6 kΩ and a calculated critical current density *J*_C_ of 72.2 ± 6.2 A cm^–2^. This is comparable to values seen by others, and in the range for transmon qubits, generally around 100 A cm^–2^.^26,27^

We turn to examination of the chemistry and structure of the JJ at the atomic scale. We conducted cross-sectional scanning transmission electron microscopy (STEM) and electron energy loss spectroscopy (EELS) on the device. The virtual annular dark field image from the 4D STEM data of the JJ, depicted in Figure 2(a), shows clear crystalline Al grains in both the top and bottom electrode. Grain sizes in the bottom electrode range between 10 and 50 nm, while the grain sizes in the top electrode are between 50 and 100 nm, with the largest grain being around 300 nm. The crystallinity and grain size is similar to those measured in JJs elsewhere.^28,29^ The oxide layer is clearly visible with a thickness between 1.6 and 1.9 nm (see Supporting Information 3).

**Figure 2:**
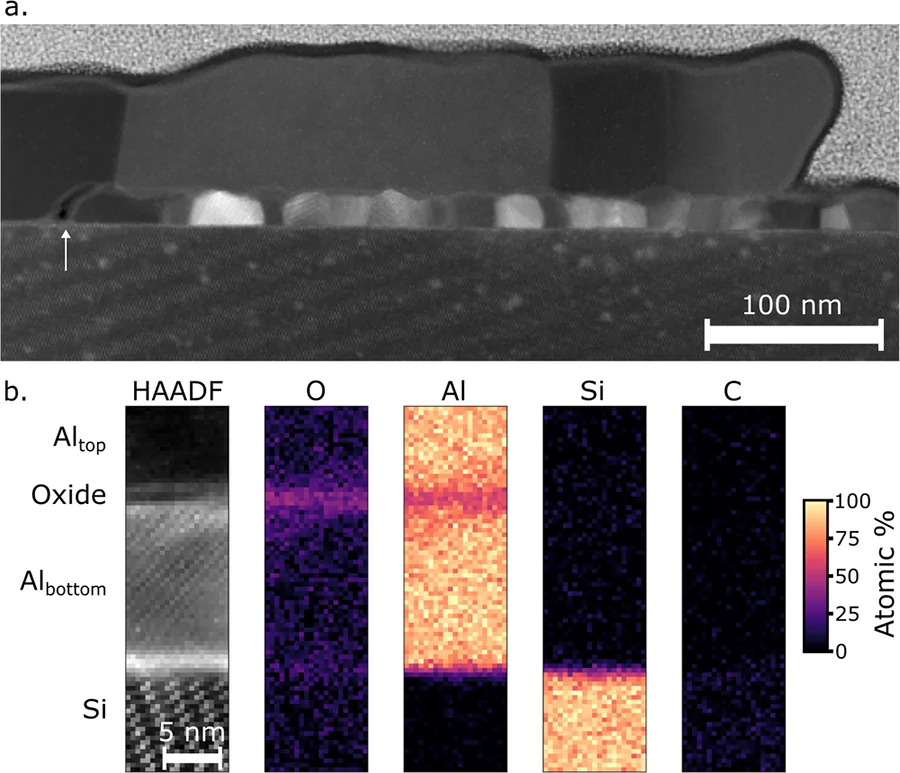
STEM/EELS. (a) Virtual annular dark field image constructed from 4D STEM data showing Al electrode microstructure in the overlap region. The void formed between the side of the bottom electrode and the climbing top electrode is indicated with a white arrow. (b) High resolution STEM-HAADF and quantitative EELS elemental maps of the device. The diffused and noisy carbon signal is due to electron beam-induced hydrocarbon contamination which forms preferentially on the more insulating substrate during data acquisition. No carbon is seen specifically concentrated at the Si/Al interface as would be expected for resist residue.

The EELS composition map, shown in Figure 2(b), examines the chemical profile within the JJ. We see that there is no evident carbon contamination within the device, either within the evaporated aluminum or any of the interfaces. The diffused, noisy carbon signal within the Si is due to electron-beam induced hydrocarbon contamination which forms preferentially on the more insulating substrate during data acquisition. There is also no oxidized silicon found on the substrate under the junction. This highlights the high cleanliness of the substrate prior to deposition, and the minimization of any residue at the Si/Al interface, which is a known source of loss in superconducting devices.^5,30^ Thus, the trench-based JJs contrast with resist-based junctions, which may exhibit non-negligible C and O signals at the interface.^7^

We note that there is a void between the side of the bottom electrode and the climbing top electrode [indicated by a white arrow in Figure 2(a)]. This is due to the deposition angle of the top electrode counter to the edge of the bottom electrode [see Figure 1(b)] which results in a shadow, similar to that seen on the nonclimbing edge of Manhattan-style junctions. We believe that the mobility of the Al atoms during deposition while the surface remains unoxidized avoids a full shadowing: the films are likely attracted to each other and the lower film appears to “bend” backward during the growth to make contact to the upper film.

We use the deposited JJs to fabricated transmon qubits. Qubits are patterned onto the chips using the Al-AlO_*x*_-Al films deposited for the junctions as the film for all features, i.e., the JJ deposition step is the only deposition in the entire fabrication process. The fabrication process is detailed in Figure 3(a) (see Supporting Information 1). The trilayer films are coated in photoresist, followed by patterning in a maskless aligner and a wet etch in aluminum etchant type A. The resist is then stripped from the film, followed by an oxygen plasma descum and a gentle vapor HF cleaning process. The vapor HF process assists with removing hydrocarbons on the surface while also removing the SiO_2_ exposed at the bottom of the trench. Figures 3(b) and (c) show an optical microscope image of an interdigitated capacitor qubit postfabrication with a capacitor gap of 5 μm.

**Figure 3:**
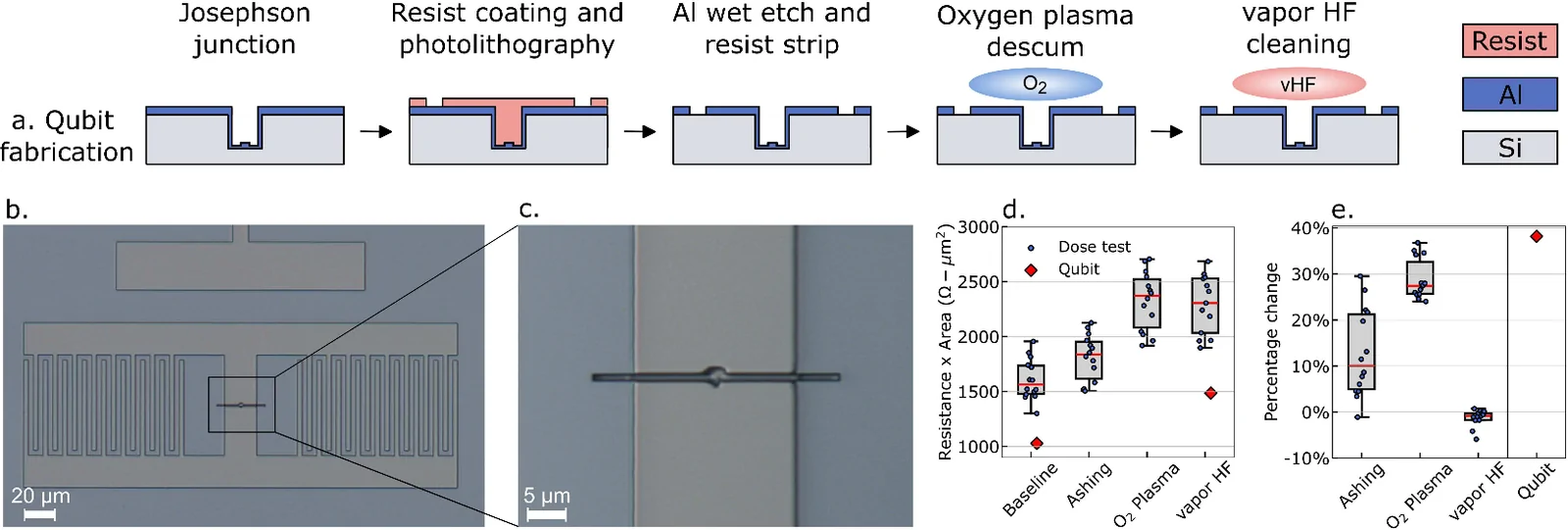
Qubit fabrication. (a) Full qubit fabrication process. The evaporated junction film is coated in photoresist, followed by photolithography and an Al wet etch. The resist is stripped from the chip, with a subsequent oxygen plasma descum and a gentle vapor HF cleaning. (b) Optical microscope image of an interdigitated capacitor qubit. (c) Magnified image of the JJ location on the qubit. (d) Room temperature junction resistance variation on a test chip after four consecutive fabrication steps: after the wet etch process and resist strip (“baseline”), after ashing, after the oxygen plasma step, and after vapor HF treatment (“vapor HF”). Red diamond indicates a test JJ on the qubit chip that was measured after wet etch and resist strip, and after completion of the full fabrication as detailed in (a). (e) Relative percentage increase in resistance from each process step. We also indicate the percentage change in resistance of the qubit throughout the entire process (baseline → qubit).

We monitored the resistance of the junction after the oxygen plasma and vapor HF processing, which is depicted in Figure 3(d). Sequential processing steps are conducted on a test chip which contains junctions of various areas post-Al wet etch and strip (indicated as “Baseline”). We first test a dedicated oxygen plasma asher (labeled “Ashing”), which results in a small increase in resistances. However, this increase is nonuniform, with a large variation over the different junction sizes [see Figure 3(e) for percentage increase per junction by each process]. The subsequent oxygen plasma descum in an Oxford reactive ion etching (RIE) system further increases the resistance (labeled “O_2_ Plasma”). In this instance, the resistance increase is significantly more consistent, around 25%–35%, in contrast to the asher, which is better for reproducibility. Finally, the vapor HF process (labeled “vapor HF”) does not appreciably affect the resistance, with the percentage being near zero. This indicates that the process is gentle enough to not damage the junction while etching the SiO_2_ and removing any surface hydrocarbons. We also conduct resonator measurements of Al samples with and without vapor HF, which is shown in Supplementary Figure S6.

We characterize five qubits on the chip, with the full characterization results shown in Table S1 (Supporting Information 5). The qubits have frequencies near 2.8 GHz (with readout resonator frequencies around 6.7 GHz), and show relaxation times (*T*_1_) and Hahn-echo dephasing times (*T*_2E_) that depend on the gap between the capacitor pads. Figures 4(a) and (b) show representative measurements of the *T*_1_ and *T*_2E_ data from the qubit with a 150 μm capacitor gap. Figures 4(c) and (d) show the qubit energy relaxation and Hahn-echo dephasing quality factors, *Q*_1_ = ω_qubit_ *T*_1_ and *Q*_2E_ = ω_qubit_ *T*_2E_, respectively, collected in an interleaved manner over several hours in 10 min intervals. Figure 4(e) shows our estimate for the pure dephasing quality factor, *Q*_φ_ = (1/*Q*_2E_ – 1/2*Q*_1_)^−1^ from consecutive *T*_1_ and *T*_2E_ measurements in the interleaved data set. We present all three coherence metrics as quality factors for ease of direct comparison (ω_qubit_ across the 5 qubits varies by ≈6.6%, see Supplementary Table S1). The pulse sequences for the respective measurements are detailed in Supporting Information 5.

**Figure 4:**
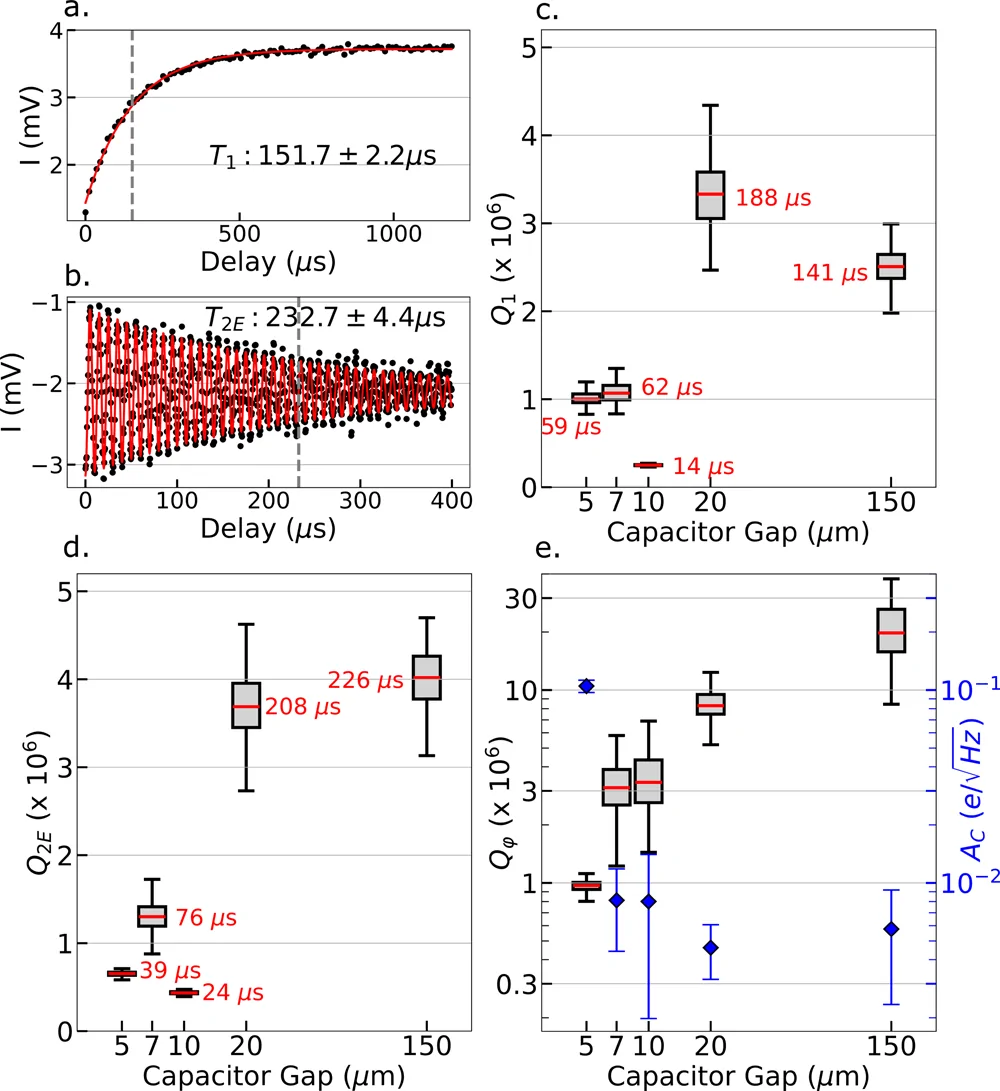
Qubit measurement data. (a, b) Representative qubit relaxation time *T*_1_ and Hahn-echo dephasing time *T*_2E_ from a qubit with a capacitor gap of 150 μm. (c, d) Qubit energy relaxation quality factor *Q*_1_ and Hahn-echo dephasing quality factor *Q*_2E_ box plots as a function of capacitor gap. Median *T*_1_ and *T*_2E_ are indicated in red, with the interquartile range (IQR) indicated with the box plot and 1.5 IQR with whiskers. (e) Qubit pure dephasing quality factor *Q*_φ_ box plot (left axis) and calculated upper bound of charge noise amplitude *A*_C_ (right axis, blue diamonds) as a function of capacitor gap.

Figures 4(c) and (d) show that the qubit quality factors are sensitive to the capacitor gap size up to a maximum median *Q*_1_ of 3.33 million. The Ramsey dephasing times (*T*_2R_) follow the same gap-dependent trend as the relaxation time, between median values of 9.5 μs for the 10 μm qubit and 69.0 μs for the 20 μm qubit (see Supplementary Table S2). This indicates that surface losses dominate the time-averaged performance for smaller gap sizes, scaling with the decrease in surface participation of the electric fields up to some threshold.^31,32^ The 150 μm qubit has a lower *T*_1_ and *Q*_1_ relative to the 20 μm qubit, which might be related to coupling to box modes or walls due to the larger extent of the electric fields.^33^ One of the qubits measured, namely the 10 μm gap qubit, does not follow the trend, and instead has low energy relaxation times for undetermined reasons. We also note that the qubits are not Purcell limited, with the limit greater than 1 ms for the qubits (see Supplementary Table S2).

The trend of the dephasing is also notable (Figure 4(e), left axis). We find that *Q*_φ_ continues to increase as the capacitor gap size increases. The low dispersive shifts and readout resonator line widths suggest that dephasing is not yet limited by shot-noise dephasing, and the transmons all have comparable *E*_J_/*E*_C_ ∼ 40–45 (see Supplementary Table S1). The ratio is lower than typical values (*E*_J_/*E*_C_ > 50), and therefore the *Q*_φ_ trend could be due to the effects of charge noise due to the higher sensitivity, alongside an effect due to the variation in *E*_J_/*E*_C_. We infer an upper bound to the charge noise amplitude *A*_C_ due to charge dispersion [Figure 4(e), right axis] by assuming that the entire *T*_φ_ contribution is charge dispersion limited (Supporting Information 6). The calculated *A*_C_ for four of the qubits is generally within margins of error, and are higher than the 10^–4^–10^–3^ $e/\sqrt{\mathrm{Hz}}$ quoted for transmons.^34^ The smallest capacitor gap qubit has a significantly higher *A*_C_, well above typical charge noise values. The calculation indicates the need for further analysis of pure dephasing to decouple other effects, such as quasiparticles^35,36^ and spectrally diffusing two-level systems (TLSs),^37,38^ from charge dispersion. We leave this subject to future study.

Understanding and quantifying *T*_1_ fluctuations is critical to moving toward scalable quantum computers.^12,39^ Therefore, conducting statistical analyses of long time traces is essential to characterizing qubit performance. These measurements were done in a second cooldown in an alternate dilution refrigerator (see Supplementary Figure S8 for the fridge wiring diagrams), with the sample remaining packaged and stored in a laboratory environment for 2 weeks. The second cooldown included a Traveling Wave Parametric Amplifier (TWPA) that enables a higher signal-to-noise ratio and consequently shorter averaging times.

Figure 5(a) shows the fluctuations of the 150 μm gap qubit (*f*_qubit_ = 2.83 GHz, *f*_res_ = 6.78 GHz) over 45 h, measured in 36 s intervals. The *T*_1_ fluctuation data of the other qubits are shown in Supplementary Figure S9. The *T*_1_ fluctuations show distinct drops in performance at various points throughout the time trace, particularly at the 25, 30, and 43 h mark. Regardless, the drops are sufficiently infrequent and shallow, resulting in the histogram following a well-defined normal distribution, with a mean *T*_1_ of 140.9 ± 18.7 μs. This is relatively close to the first cooldown, which signifies reproducible performance over different cooldowns and different dilution refrigerators.

**Figure 5:**
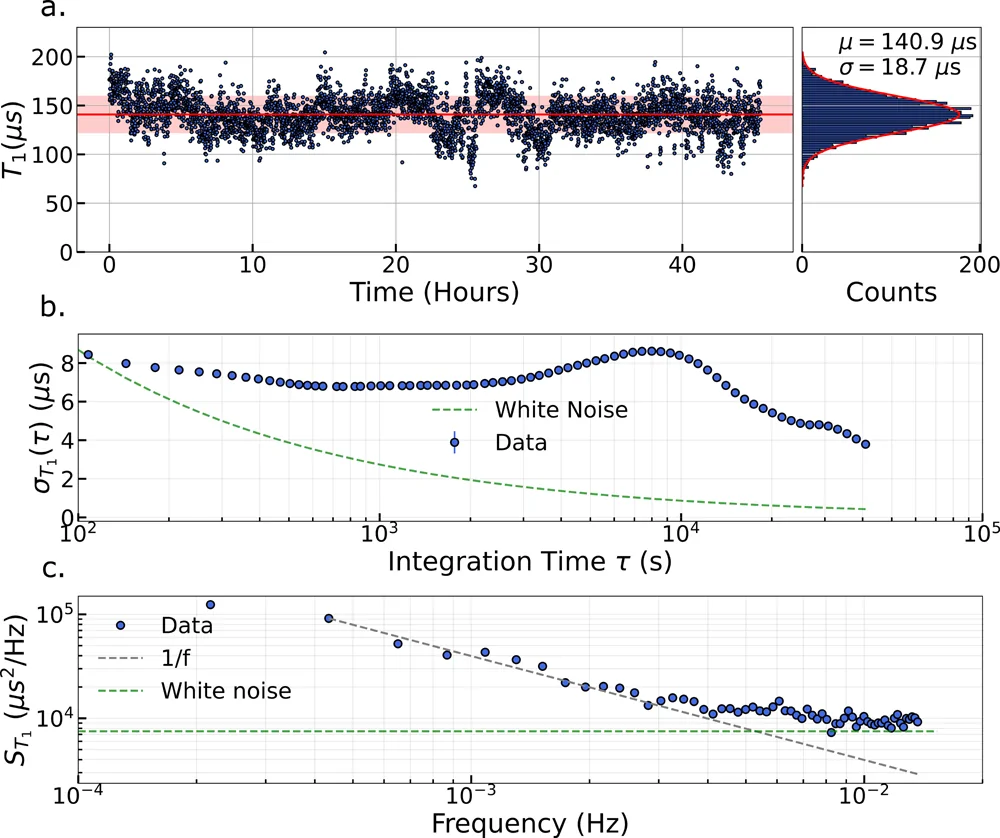
*T*_1_ fluctuation analysis of the 150 μm gap qubit (*f*_qubit_ = 2.83 GHz, *f*_res_ = 6.78 GHz) from cooldown 2. (a) Fluctuation data taken over 45 h in 36 s intervals. Histogram showing normal distribution with a mean of 140.9 ± 18.7 μs. (b) Overlapping Allan deviation plot. (c) Spectral density calculated using the Welch method. Note that dashed lines indicate the shape of the respective noise within each analysis and are purely illustrative, not fits.

Similar to Burnett et al.^39^ and Berritta et al.,^12^ we conduct statistical analyses, specifically the overlapping Allan deviation^40^ and the spectral density, of the *T*_1_ fluctuations. The overlapping Allan deviation [Figure 5(b)] shows a relatively clear plateau until the peak around τ = 10^4^ s. We note that the Allan deviation is around 7.5 μs, significantly lower than the value measured in a qubit which uses a resist-based JJ with a comparable *T*_1_ in ref 12 (mean *T*_1_ ≈ 168 μs, with lowest σ_*T*_1__ ≈ 13 μs), signifying notably lower fluctuations within the device. The peak at τ = 10^4^ s is likely due to a Lorentzian noise process, since no power-law noise processes produce a peak.^12,40^ Due to the long-range nature of the correlation and relatively shallow increase, this peak could be due to some weakly coupled TLSs. The origin of this peak is difficult to conclude at this time.

The power spectral density (PSD) fluctuation is extracted using the Welch method,^41^ and shown in Figure 5(c). The trend corroborates the data from the Allan deviation, showing that the noise is primarily 1/*f*-like, with the saturation at higher frequencies attributed to reaching a white noise floor. The lack of any significant deviation from the 1/*f* slope within the spectral density indicates that there is no single, strongly coupled TLS that is switching to near the qubit frequency within the range of noise frequencies scanned. This lack of a single significant switching event is true for four of the five qubits. The 20 um gap qubit has a fluctuation in relaxation and dephasing rates that is indicative of a strongly-coupled TLS. This indicates that while the trench-based qubit might improve fluctuations, a more comprehensive analysis over more devices and longer time horizons is necessary. Remarkably, we find that *T*_2E_ fluctuations follow closer to a white noise distribution. More details on the fluctuation analysis are contained in Supporting Information 8.

Examining the results, we observe that the quality factors of the qubits fabricated using our scheme, while not yet reaching the current state-of-the-art (SOTA),^3,8^ are nevertheless comparable to earlier iterations of resist-based qubit fabrication methods.^37,42^ With the integration of previously established fabrication improvements, the design has the potential to approach SOTA performance. Although we have not fully established a measurable advantage in terms of the maximum achievable performance in comparison to conventional Dolan-bridge junctions, we have observed higher quality factors than seen with other novel junction fabrication schemes demonstrated so far.^19,21–23^

The *T*_1_ fluctuations of the qubits measured here are lower than one would expect for a comparable qubit *T*_1_,^12,43^ as calculated by the Allan deviation. To compare against a larger number of works, we utilize the robust coefficient of variation, RCV (defined as the ratio of the interquartile range to the median), as a useful yet imperfect metric for comparison.^44^ Our RCV remain below 0.2, while other works are usually 0.2–0.3 and beyond^5,7,8,45^ (see Supporting Information 9). We also compare the range normalized by the median and find it to be lower as well. Such long-term stability of qubit coherence is desirable for larger-scale qubit chips, improving reproducibility and reducing the need for frequent qubit recharacterization.

There are several avenues available to further improve the fabrication of the qubits shown here. This involves exploring alternative etch processes, both for the silicon trench and the aluminum film, and further cleaning processes. Additionally, the lack of an overetch of the Si due to the use of an Al wet etch makes these devices unable to leverage the reduction in the participation of the lossy interfaces, which could further improve qubit performance, particularly for the more compact designs.^32,46,47^ Therefore, the performance shown here is primarily for initial benchmarking of the process, indicating proof-of-principle demonstration of the viability of the scheme in producing high-quality qubits.

A significant consideration in the design and integration of the new JJ design is the ability to integrate the trench-based junction into any existing Si-based planar qubit fabrication process. The specifics of the design allow for the Si to be etched postbase layer deposition, followed by deposition of the JJ and subsequent etch of the non-JJ film. The simplicity of the incorporation enables the use of this design in conjunction with improvements in base layer fabrication improvements that have been found in recent years.

A key feature of the junction design described here is the substantial widening of the process window for JJ fabrication. The design is significantly more material-agnostic in comparison to resist-based junction fabrication schemes, only limited by the ability of a given material to be directionally evaporated. The material-agnosticism opens the door to an extensive exploration of the interplay between alternative materials with qubit performance, without the limitation of the damage seen in traditional trilayer junction fabrication schemes.

We also note that the substrate itself is also not limited to Si. The primary requirement is the presence of smooth sidewalls and bottom postetching to prevent climbing issues and variation in junction areas. Thus, the fabrication approach can be conducted on substrates such as sapphire, MgO, and others, with appropriate etch chemistries for the respective materials. This method can also be conducted on sufficiently thick metal films to create the deep trenches needed.

Overall, this fabrication scheme provides new avenues of performance and materials investigation that was previously unexplored due to limitations of existing processes. The design shows promise for integration to existing qubit fabrication processes, while removing the hindrances of resist-based and other fabrication methods. We anticipate future studies involving approaches to substrate annealing and cleaning, integration with high-performing base layers,^3,8,48^ and detailed noise spectroscopy,^49^ all in conjunction with alternative JJ materials to explore new avenues to improve qubit performance.

In conclusion, we have developed a novel Josephson junction fabrication scheme that is more material-agnostic and offers substantially wider thermal and chemical process windows due to avoiding the use of a polymer liftoff mask. The method is scalable due to the use of standard silicon etching, and integrable with existing semiconductor and superconductor process technology alongside Josephson junction shadow-evaporation tools for wafer sizes up to 200 mm. We fabricate qubits using this scheme and measure qubits with the highest median *T*_1_ of 188 μs and *Q*_1_ of 3.33 million. We see minimal interface contamination and find capacitor gap-dependent performance, indicating a surface-participation-ratio limited device which may be improved further through additional postprocessing. Coherence fluctuation analysis using Allan deviation and PSD show that the deviation in the performance is lower compared to other comparable qubits. Future work will involve the use of alternative electrode and tunnel barrier materials, integration with nonaluminum base layers, and exploration of cleaner fabrication processes and thermal annealing steps.

## Supplementary Material



## Data Availability

The data that support this article is openly available at 10.5281/zenodo.19303492.^50^

## Notes

Further details about the following sections: (1) device fabrication; (2) room temperature JJ measurement; (3) chemical and interface characterization; (4) vapor HF effects on Al resonators; (5) qubit design and measurement; (6) Purcell limit, dephasing, and charge noise; (7) fridge wiring diagrams; (8) detailed *T*_1_ and *T*_2E_ fluctuation analysis; and (9) robust coefficient of variation comparison (PDF)
