# Supplementary material for: Resist-Free Shadow Deposition Using Silicon Trenches for Josephson Junctions in Superconducting Qubits
Source: Nano Lett. 2026 Oct 22;26(43):14845–52. doi: 10.1021/acs.nanolett.6c02734 (PMC13741901; doi:10.1021/acs.nanolett.6c02734)
Supplement: Supplementary file 1 [file nl6c02734_si_001.pdf]

Supplementary Information: Resist-free shadow deposition using silicon trenches for Josephson junctions in superconducting qubits

Tathagata Banerjee,[1,*] Stephen Daniel Funni,[2] Saswata Roy,[3] Judy J. Cha,[2] and Valla Fatemi[1,†]

[1]*School of Applied and Engineering Physics, Cornell University, Ithaca, NY 14853, USA*
[2]*Department of Materials Science and Engineering, Cornell University, Ithaca, NY 14853, USA*
[3]*Department of Physics, Cornell University, Ithaca, NY 14853, USA*

1. DEVICE FABRICATION

Trench fabrication

We begin by taking an intrinsic, high resistivity ($> 10,000\ \Omega$-cm) undoped, single-side-polished, float zone four-inch Si (100) wafer sourced from WaferPro, and oxidize using a dry oxidation process (no HCl) at 1000 °C for 20 min to grow approximately 170 nm of SiO_2 on the wafer. The oxidized wafer is then spin-coated at 3000 rpm with Kayaku 495k PMMA A8 e-beam resist and baked at 170 °C for 15 min. The measured thickness of the resist is 378 ± 4 nm. The wafer is then spin-coated with DisCharge anti-charging agent from DisChem Inc. at 2000 rpm to prevent pattern distortion due to charge accumulation in the insulating SiO_2 during lithography.

Electron-beam lithography is conducted in a JEOL 6300 series system with a beam current of 10 nA with a dose of $1000\,\mu C\,cm^{-2}$. Post lithography, the wafer is rinsed in deionized water (DI H_2O) to remove the DisCharge coating. The pattern is developed at room temperature in a 1:3 MIBK:IPA (methyl isobutylketone:isopropanol) for 1 min, followed by a 30 s rinse in IPA and blow drying with nitrogen.

SiO_2 etching is conducted in an Oxford PlasmaLab 100 Inductively Coupled Plasma (ICP) System using a CHF_3/O_2 based process for 1.5 min (CHF_3: 52 sccm, O_2: 2 sccm, ICP: 2500 W, RIE: 15 W, pressure: 5 mTorr). The chamber was prepared with a 10 min oxygen plasma clean and a 5 min chamber seasoning with a blank Si wafer prior to etching. The e-beam resist is then stripped from the surface in an Integrated Micro Materials AZ300T stripper bath at 70 °C for 15 min. This is followed by two consecutive sonication baths in IPA for 15 min and 5 min respectively.

The Si is etched with the SiO_2 hard mask pattern in an HBr-based Oxford PlasmaPro NGP100 ICP-RIE etcher for 3.5 min (HBr: 20 sccm, ICP: 2000 W, RIE: 40 W, pressure: 8 mTorr). The etcher was prepared with a 10 min SF_6 plasma clean and a 10 min chamber seasoning with a blank Si wafer prior to etching. Following the etch, the measured

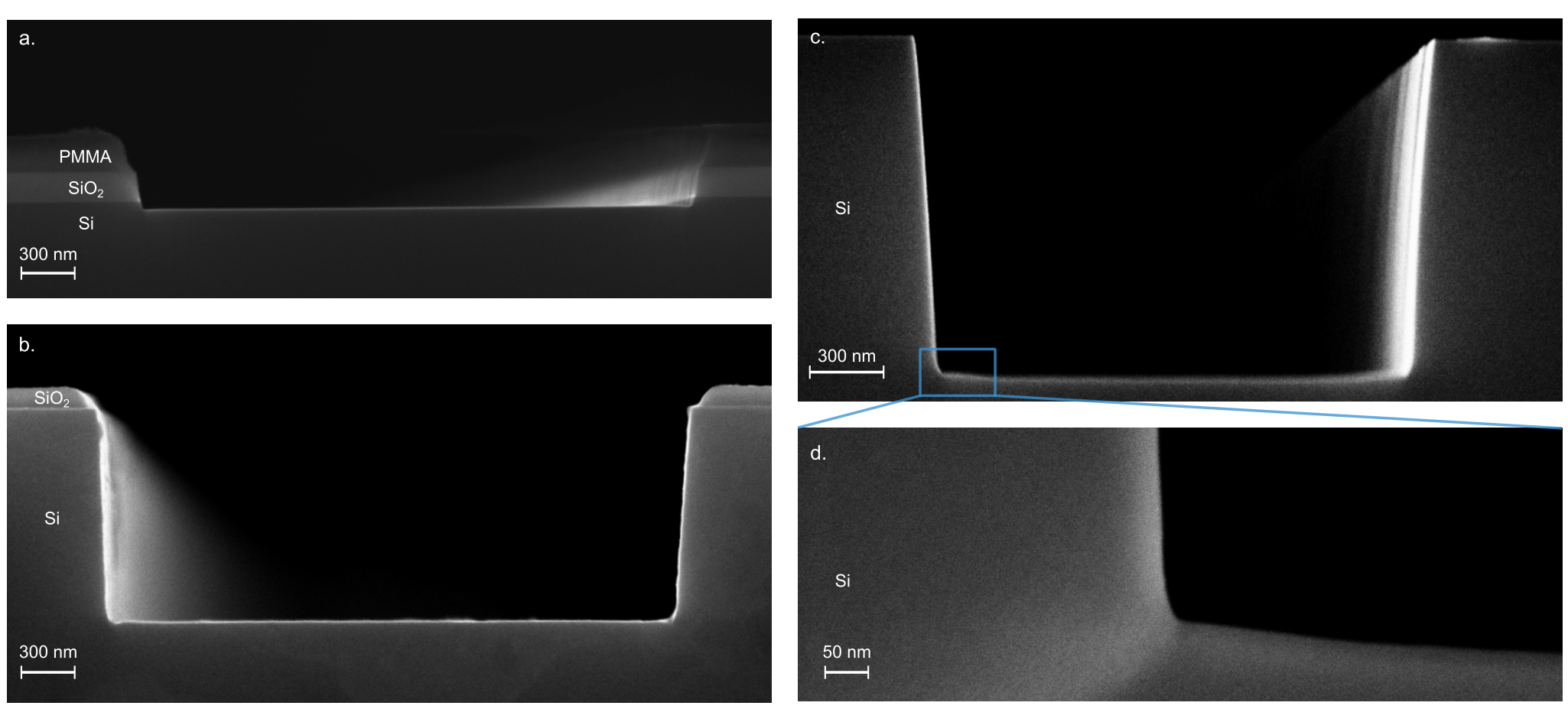

FIG. S1. Cross-sectional SEM of respective fabrication steps during trench fabrication. (a) After SiO_2 etching in CHF_3/O_2, (b) after Si etching in HBr, (c) after SiO_2 strip in HF, and (d) an enlarged image of the trench corner.

* tb548@cornell.edu
† vf82@cornell.edu

thickness of the remaining hard mask is 97 ± 4 nm, with a Si trench depth of approximately 1.1 µm and a sidewall angle of approximately 85°-88°. The remaining SiO_2 hard mask is stripped by soaking in a room temperature 10:1 hydrofluoric acid (HF) bath (Transene Company, Inc.) for 5 min followed by a rinse in DI H_2O for 30 s and blow drying with air. Cross-sectional SEMs after various etching steps are shown in Fig. S1.

For dicing, the wafer is coated with Microposit S1813 at 4000 rpm and baked at 90 °C for 60 s. The wafer is then diced into 7.5 mm×7.5 mm pieces using a DISCO dicing saw, followed by a resist stripping process similar to the one conducted post-SiO_2 etch: the pieces are soaked in an AZ300T stripper bath at 70 °C bath for 15 min followed by two consecutive 15 min IPA sonication baths and blow drying with air.

Josephson junction fabrication

Immediately prior to JJ deposition, the etched sample is soaked in a 10:1 buffered oxide etch (BOE) solution (Transene Company, Inc.) for 60 s to remove any remaining resist residue and SiO_2. The samples are then rinsed in DI H_2O for 30 s followed by blow drying with air.

The samples are loaded into an Angstrom Quantum Series electron beam evaporation system, which is equipped with separate evaporation and oxidation chambers. While in the loadlock, the oxidation chamber is flushed with 1 Torr oxygen for 10 s three consecutive times to flush any contamination from the oxygen lines. The base pressure prior to evaporation is below 2×10^{-8} Torr.

We evaporate 5N purity aluminum from a copper hearth without a crucible. No ion milling is conducted prior to deposition. The sample is first tilted to an angle of 49°, after which Al for the first electrode is deposited at $2\,\text{Å}\,\text{s}^{-1}$ until a nominal thickness of 30.5 nm. The Al is oxidized at 1 Torr for 5 min using 100% O_2, followed by the deposition of the second electrode at a $-49°$ angle. The second electrode is also evaporated at $2\,\text{Å}\,\text{s}^{-1}$ until it reaches a nominal thickness of 123 nm. The true thicknesses of the two electrodes are 20 nm and 80 nm respectively, due to the lower surface flux from the tilted angle. We compare the fabrication process of conventional Dolan-bridge JJs to trench-based JJs in Fig. S2.

Qubit fabrication

The chip with the evaporated film and the Josephson junctions is spin-coated with HMDS-P20 primer (Transene Company, Inc.) at 2500 rpm followed by a coating of Microposit S1813 photoresist at 4000 rpm. The resist is then baked at 110 °C for 1 min. The pattern is exposed in a Heidelberg Instruments MLA 150 Advanced Maskless Aligner with a dose of 110 mJ and a defocus of -2 (−18 µm). The pattern is developed in room temperature AZ726 MIF for 60 s followed by a rinse in DI H_2O for 30 s and blow drying with air.

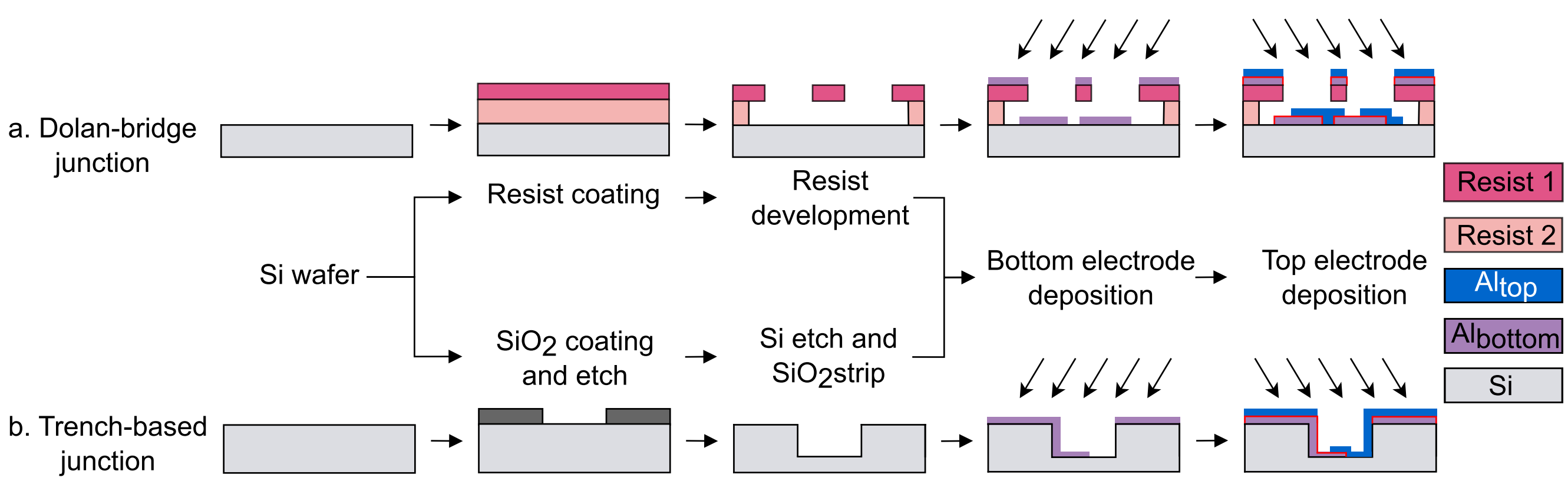

FIG. S2. Josephson junction fabrication process. (a) Dolan-bridge junction fabrication: a wafer is coated with a bilayer e-beam resist stack, followed by lithography and development. This is followed by double angle deposition with an intermediate oxidation step to form the JJ. Finally, the junction undergoes a liftoff process to remove the resist (not shown). (b) Trench-based junction fabrication: a silicon wafer is thermally oxidized to form SiO_2, followed by lithography and etching to form the hard mask. The Si is dry-etched followed by removal of the hard mask using hydrofluoric acid. This is followed by a double angle deposition with an intermediate oxidation step to form the overlap.

The pattern is wet-etched into the Al film with a 1.5 min soak in room temperature Aluminum etchant - type A (Transene Company, Inc.), composed of 40-80 % phosphoric acid, 3-20 % acetic acid and 1-5 % nitric acid. The wet etch is followed by soaking the sample in DI H_2O for 30 s and blow drying with air.

The resist is stripped from the chip through subsequent sonication in acetone and IPA for 5 min each and blow drying with air. We then remove resist residue using a 1 min O_2 plasma descum process (75 W, O_2: 50 sccm, pressure: 60 mTorr,) in an Oxford PlasmaLab 80+ RIE system. Finally, the chip undergoes treatment in vapor hydrofluoric acid using a Primaxx µEtch system. The process begins with a 15 min *in-situ* drying process at 45 °C, with vaporized ethanol (300 sccm) and nitrogen gas (1500 sccm) at a pressure of 90 Torr. This is immediately followed by the 1 min vapor HF process (vHF: 190 sccm, ethanol: 210 sccm, N_2: 1425 sccm). The chamber is then purged three times with nitrogen before venting. The chip is then wire bonded, packaged, and loaded into the cryostat for measurement.

2. ROOM TEMPERATURE JJ MEASUREMENT

We conduct dose testing of Josephson junctions (JJ) fabricated using the Si-trench scheme with a variety of oxidation doses and areas. Dose testing data of the JJs are shown in Fig. S3. Room-temperature two-probe measurements are conducted on all junctions using an Everbeing EB-6 high precision probe station and a Keithley 4200A-SCS Parameter Analyzer. The measured resistance between probe tips is less than 3 Ω.

We estimate the critical current density $J_c = I_c/A$ with the Ambegaokar-Baratoff formula [1],

$$I_c = \frac{\pi\Delta}{2eR_n}, \tag{1}$$

where I_c is the critical current, A is the junction area, R_n is the normal state resistance, and we choose $\Delta = 180\,\mu\text{eV}$ to represent the superconducting energy gap. Combining the J_C and I_C formula, we find;

$$J_c = \frac{I_c R_n}{R_n A} = \frac{\pi\Delta}{2eR_n A}, \tag{2}$$

$$J_c = \frac{282.7\mu\text{V}}{R_n A}, \tag{3}$$

where $R_n A$ is simply the resistance-area product of the Josephson junction. The calculated J_C of the various doses is shown in Table S1. The J_C is similar to junctions measured elsewhere [2]. The area used for qubit fabrication, 360 nm×180 nm, is depicted with a dashed black line in Fig. S3.

Fig. S3 shows that the resistance-area relation follows the expected trend, $R_n \propto 1/A$, across a range of areas, between 0.02 µm^2 and 0.42 µm^2. This indicates that the junction area is stable and the etch pattern does not undergo significant warping or variation as the lateral dimensions increase by a factor of ≈ 5.

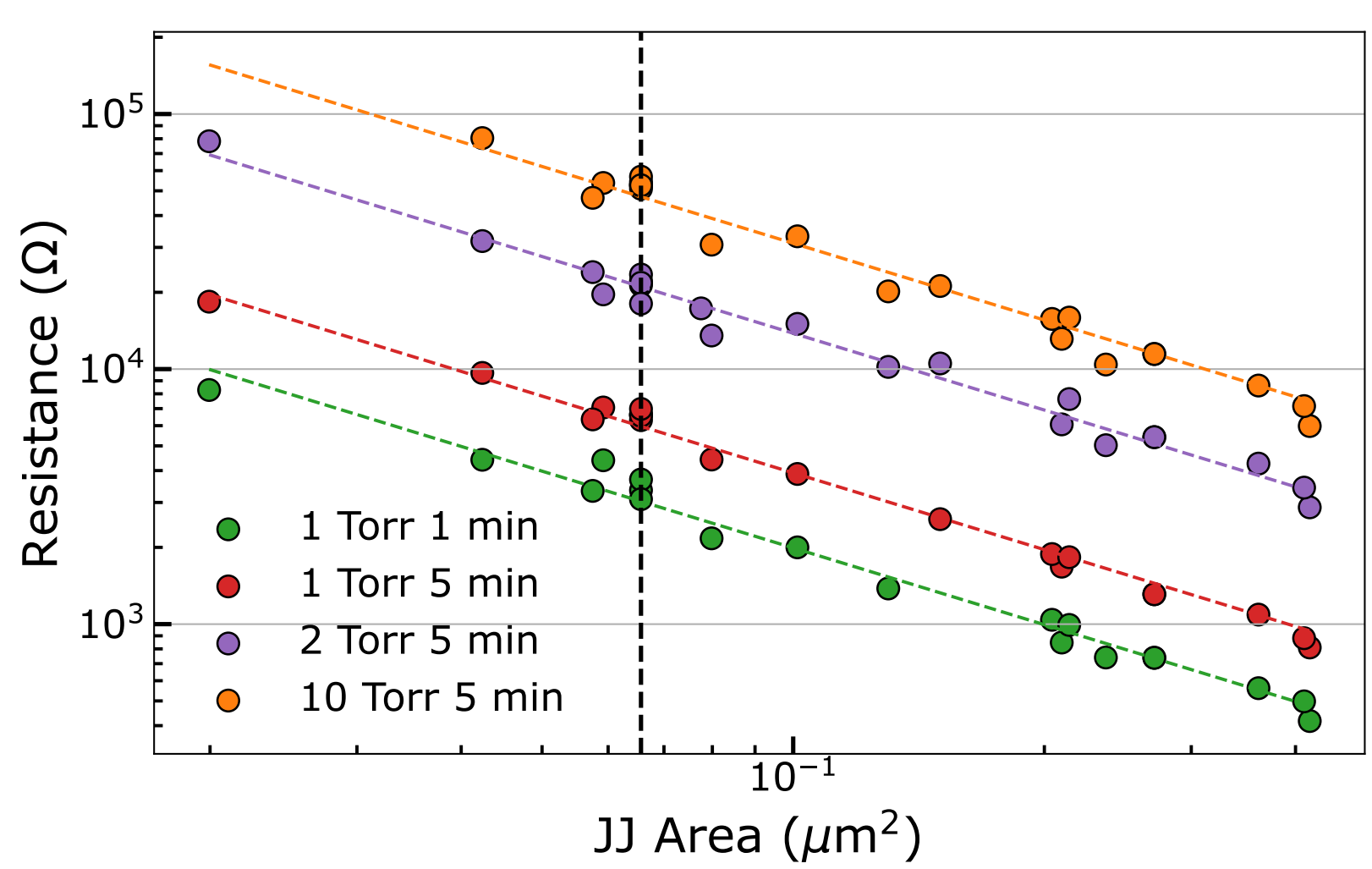

FIG. S3. Oxidation dose testing. We conduct four different oxidation doses on test chips containing a variety of JJ areas to examine J_C and the scaling. Lines indicate the mean resistance-area product. Black line indicates the junction area for the device shown in Fig. 1 and the qubits in Figs. 4-5.

Pressure	Time	Mean R_nA	J_C
1 Torr	1 min	$199.1 \pm 24.2\ \Omega\,\mu m^2$	$142.1 \pm 17.3\ A\,cm^{-2}$
1 Torr	5 min	$391.8 \pm 33.5\ \Omega\,\mu m^2$	$72.2 \pm 6.2\ A\,cm^{-2}$
2 Torr	5 min	$1381.6 \pm 152\ \Omega\,\mu m^2$	$20.5 \pm 2.3\ A\,cm^{-2}$
10 Torr	5 min	$3114.3 \pm 382\ \Omega\,\mu m^2$	$9.1 \pm 1.1\ A\,cm^{-2}$

TABLE S1. Measured mean resistance-area product R_nA and calculated critical current density J_C of the JJs from oxidation dose testing.

3. FURTHER CHEMICAL AND INTERFACE CHARACTERIZATION

Electron transparent cross-sectional samples were prepared for STEM with a Thermo-Fisher Helios G4-UX Dual-Beam SEM/FIB with final thinning at 2 kV. A Thermo-Fisher Scientific (TFS) Spectra S/TEM operating at 300 kV with a 21.4 mrad convergence angle and a beam current of 46 pA was used for all experiments. High angle annular dark field (HAADF) imaging was acquired with a detector collection range of 48–200 mrad. 4D STEM data was collected on an EMPAD direct electron detector using a 1 ms dwell time per pixel. The virtual annular dark field image was formed from the 4D STEM data set by integrating the intensity in each diffraction pattern over the angular range of 23-35 mrad, which maximized the diffraction contrast.

EELS data was collected using a TFS Iliad spectrometer with Zebra detector [3]. The EELS acceptance angle was 51.6 mrad and the dispersion was 423 meV/channel. All STEM data analyses, including quantitative EELS mapping, were performed using the python package SingleOrigin [4].

Fig. S4(a) shows the virtual annular dark field image, identical to the one shown in Fig. 2(a) with a color map applied. Included are select diffraction patterns from 4D STEM [Fig. S4(b)]. Crystalline grains show Kikuchi lines and/or shadow lattice images. We see that all grains except the two amorphous layers – the AlO_x tunnel barrier and the evaporated carbon from the FIB preparation – show either indication of crystallinity.

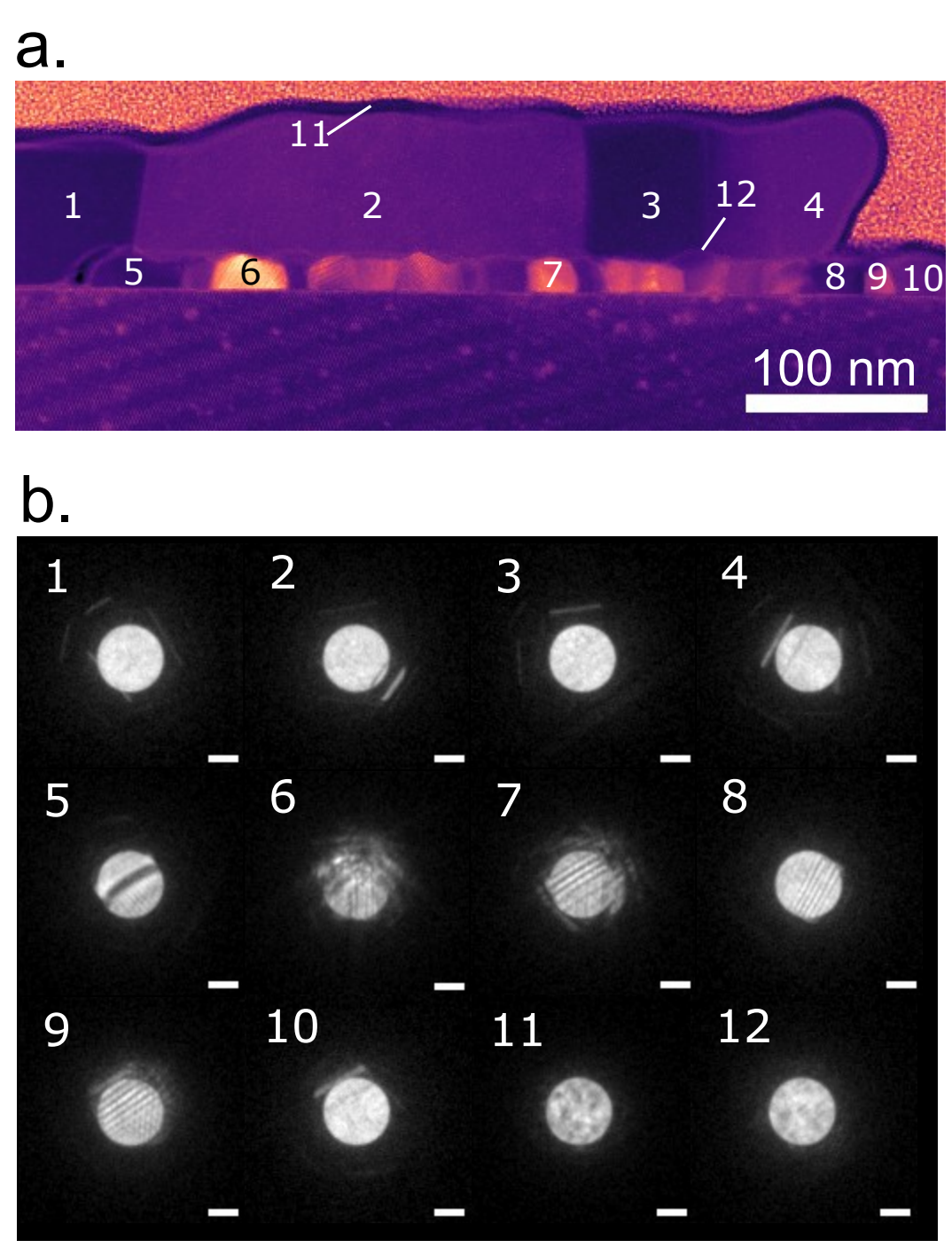

FIG. S4. Extended STEM/EELS data. (a) Virtual annular dark field image (same as Fig. 2(a) but with colormap applied) (b) Selected convergent beam electron diffraction (CBED) patterns showing crystallinity of Al electrode grains. Note Kikuchi lines and/or shadow lattice images in 1-10, indicating crystalline order. Patterns 11 and 12 are from amorphous materials: evaporated carbon (deposited prior to FIB sample preparation) and AlO_x junction barrier, respectively. These two patterns lack crystalline signatures seen in the Al metal electrodes. The mottled contrast in the bright field disk is also indicative of amorphous order. CBED scale bars are 20 mrad.

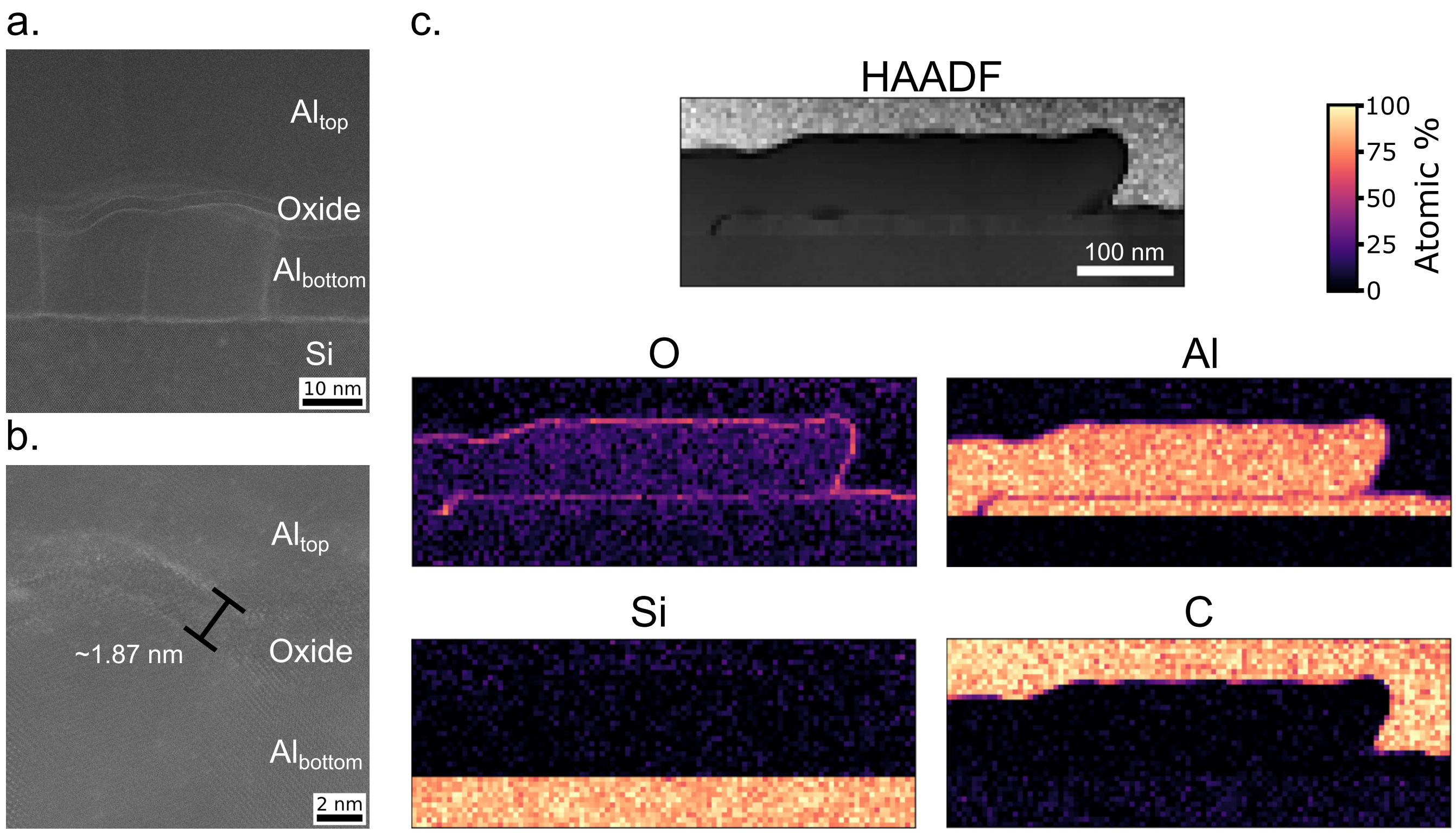

FIG. S5. (a) STEM-HAADF image of device interfaces. The near zone axis grain in this image is highly crystalline and corresponds to grain 6 in Fig. S4. (b) STEM-HAADF image of the oxide layer. (c) EELS mapping of elemental composition of the oxide barrier and Al/Si interfaces processed the same way as in Fig. 2(b). Note the lack of carbon contamination at the substrate interface.

Fig. S5(a) and (b) show a zoomed in cross-section. The grains and the oxide barrier are clearly visible. The oxide thickness is estimated to be around 1.9 nm. Beyond the center of Fig. S5(b), we see that the oxide barrier seems to diverge. This is an artifact of the projection and the roughness of the bottom electrode. It is likely that the apparent divergence is due to the oxide growth on two different grains or the different facets of the same grain.

Fig. S5(c) shows elemental mapping identical to Fig. 2(b), but for the entire junction cross-section. The enlarged view corroborates the observation that there is minimal carbon contamination at the interfaces or within the film. The oxygen is localized to the the tunnel barrier, with no oxide present at the Si/Al interface. The diffused background signal for the carbon and oxygen is a result of the imaging process (hydrocarbon contamination) and sample preparation (air exposure after FIB preparation) respectively. Any carbon or oxygen intrinsic to the as-prepared device is not detectable in this data below the $\approx 5\%$ level due to masking by these artifacts.

4. AL RESONATOR DATA

We measure Al resonators to examine if the vapor HF impacts performance, shown in Fig. S6. The fabrication is similar to the qubit chip, with the primary difference being the use of only a single Al film 60 nm thick, and patterning of the resonators using a g-line stepper. The pattern used is from the Boulder Cryogenic Testbed, which contains eight branches on each chip with target frequencies between $4-8$ GHz [5]. We see no measurable difference between the samples with and without the vapor HF process.

5. QUBIT DESIGN AND MEASUREMENT

The qubit design was adapted from a design shared with us by Parrott, McFadden, and McRae at NIST-Boulder. We show a section of the GDSII of the chip in Fig. S7. The chip contains three different designs of capacitor pads – rectangular, interdigitated, and paddles. The paddle capacitor has the largest capacitor gap of 150 µm (Q-150),

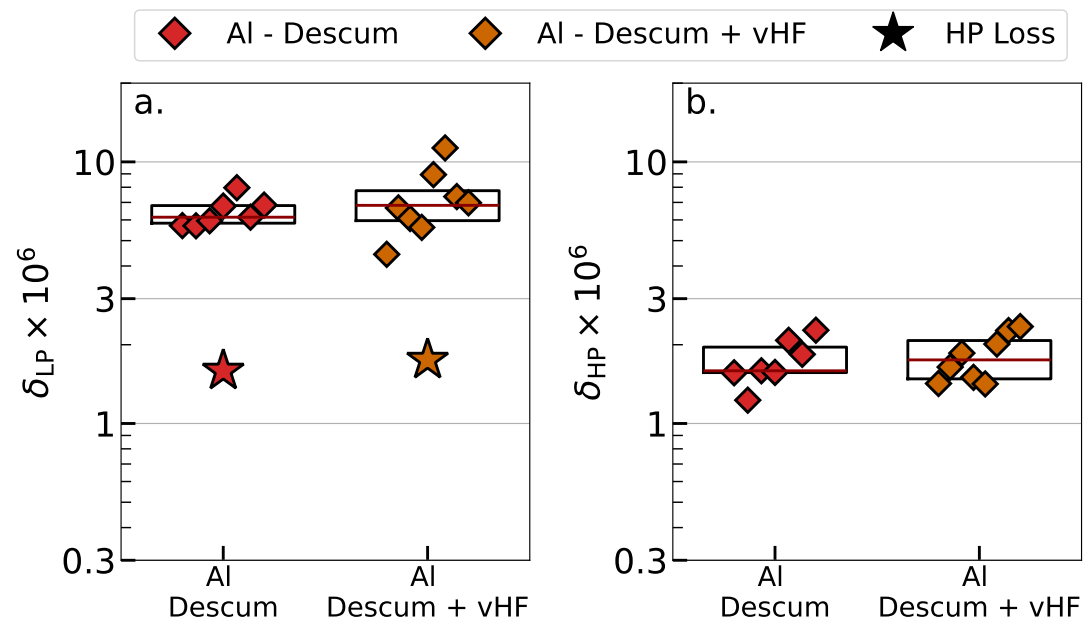

FIG. S6. Al resonator data. (a) low power loss (δ_{LP}) and (b) high power loss (δ_{HP}) for an Al resonator processed similarly to the qubit chip, with and without the vapor HF step. We see that the vapor HF does not affect the resonator loss in any meaningful way.

followed by the rectangular pad capacitor with a gap of 20 µm (Q-20). The three smaller qubits measured – 5, 7, and 10 µm gaps (Q-5, Q-7, and Q-10) – are interdigitated capacitors.

There is no independent drive line for the qubit, instead it is driven through the feedline and the readout resonator. There are also patterned square 8 µm×8 µm flux traps on the chip away from the resonators and capacitors to trap stray vortices. The nearest patterned traps are at least 100 µm away from the major patterned features.

The full characterization details of all 5 qubits is shown in Table S2. The characterization was conducted during the first cooldown. In the second cooldown, f_{res} changed less than 100 kHz, qubit frequencies were within 100 MHz of the first cooldown (generally moved lower), and T_1 times did not change more than 20%. We note that the suppression of the coherence time of Q-150 relative to Q-20 has been internally observed in other samples made with standard resist-based JJs and qubits with different base layers, indicating that this is not related to the scheme we have presented here.

Qubit measurement

The qubit is measured in two different Bluefors LD250 dilution refrigerators, both with base temperatures below 10 mK. The control and the readouts in both cooldowns are conducted using the same Quantum Machines OPX+ instrument, in conjunction with a Quantum Machines Octave instrument. Control pulses are generated in the OPX+ and up-converted using local oscillators (LO) and IQ mixers present within the Octave. Pulses are digitally triggered, with a trigger buffer on either side of the pulse. Measurements were conducted with a readout length of 5 µs. The

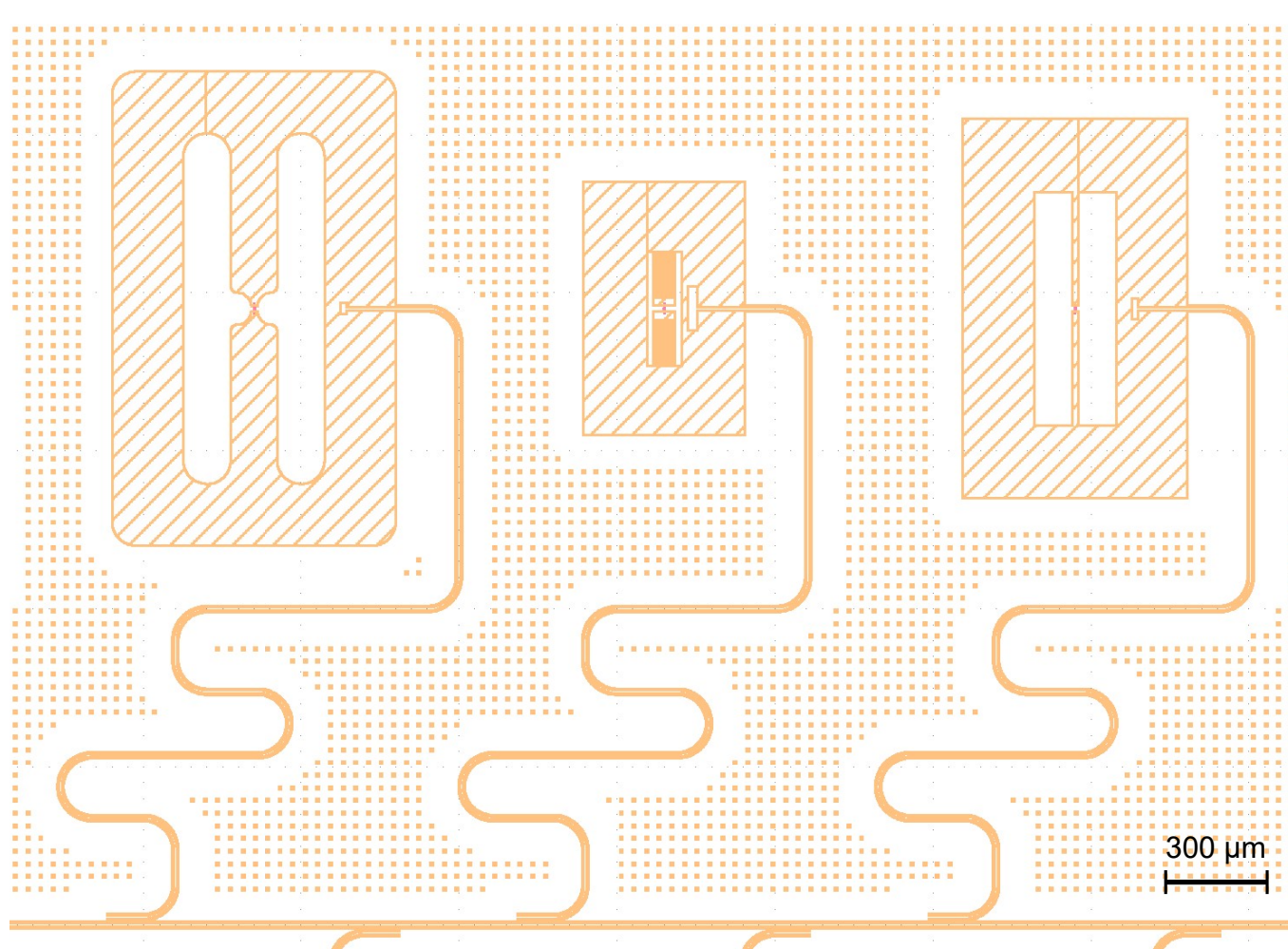

FIG. S7. GDSII image of as section qubit chip. We see the three types of qubit capacitors present in the design – rectangular pad capacitor (Q-20), interdigitated capacitor (Q-5, Q-7, and Q-10), and paddle capacitor (Q-150). The qubits are driven through the feedline. The design contains flux traps to trap stray magnetic vortices.

Qubit	Q-5	Q-7	Q-10	Q-20	Q-150
Capacitor gap	5 µm	7 µm	10 µm	20 µm	150 µm
f_{res}	6.62 GHz	6.73 GHz	6.80 GHz	6.65 GHz	6.78 GHz
f_{qubit}	2661.0 MHz	2714.2 MHz	2838.1 MHz	2811.6 MHz	2828.0 MHz
T_1	60.4±4.6 µs	66.0±13.8 µs	14.1±0.5 µs	187.9±27.1 µs	141.2±11.6 µs
T_{2R}	24.1±4.3 µs	22.0±8.5 µs	9.5±3.3 µs	69.0±24.6 µs	48.9±22.8 µs
T_{2E}	39.0±1.8 µs	76.1±9.4 µs	24.3±1.1 µs	208.3±23.5 µs	225.7±19.0 µs
$\chi/2\pi$	−90 kHz	−110 kHz	−50 kHz	−70 kHz	−70 kHz
$-\alpha/2\pi$	168 MHz	190 MHz	198 MHz	190.5 MHz	177 MHz
$\kappa_{\text{res}}/2\pi$	181 kHz	206 kHz	204 kHz	667 kHz	278 kHz
$\Delta/2\pi$	-3959.0 MHz	-4015.8 MHz	-3961.9 MHz	-3838.4 MHz	-3952.0 MHz
$g/2\pi$	93.6 MHz	98.9 MHz	64.5 MHz	75.4 MHz	80.3 MHz
T_1^P	17.0 ms	13.6 ms	29.8 ms	6.1 ms	109.3 ms
T_φ	57.7±4.1 µs	200.0±83.6 µs	219.1±160.8 µs	495.7±124.9 µs	1278.4±665.7 µs
E_J/E_C	46.9	39.8	40.0	41.9	47.5
A_C	104.8×10^{-3} e/$\sqrt{\text{Hz}}$	8.1×10^{-3} e/$\sqrt{\text{Hz}}$	8.0×10^{-3} e/$\sqrt{\text{Hz}}$	4.6×10^{-3} e/$\sqrt{\text{Hz}}$	5.8×10^{-3} e/$\sqrt{\text{Hz}}$

TABLE S2. Measured and calculated parameters of the 5 qubits on the chip during the first cooldown. Measured parameters: f_{res}: resonator frequency, f_{qubit}: qubit frequency; T_1: qubit relaxation time; T_{2R}: Ramsey dephasing time; T_{2E}: Hahn-echo dephasing time; χ: dispersive shift; $-\alpha$: anharmonicity; κ_{res}: resonator energy decay rate. Calculated parameters: $\Delta/2\pi = f_{\text{qubit}} - f_{\text{res}}$: detuning; g: resonator-qubit coupling strength; T_1^P: Purcell limit; T_φ: pure dephasing time; E_J/E_C: ratio calculated via Hamiltonian numerical diagonalization; A_C: charge noise amplitude. All values where applicable are reported in linear frequencies (kHz, MHz, or GHz).

π-pulse had a length of 1 µs and was shaped like a gaussian, terminated at 5σ.

Relaxation time T_1 was measured by sending a π-pulse followed by a time delay τ before measurement. The waiting time between consecutive measurements was set to at least $6T_1$ to ensure the qubit was depopulated before the subsequent measurement. The resulting decay was by a standard exponential with an offset, $Ae^{-\tau/T_1} + C$[6].

The Hahn-echo measurement to determine T_{2E} was conducted by sending a pulse sequence of $\pi/2 - \pi - \pi/2$ with a $\tau/2$ delay between the pulses with zero detuning from qubit frequency. The second $\pi/2$-pulse has a phase offset, increasing with each delay time. A large phase increment ensures the timescale of the decay and oscillations are well separated. The resulting oscillations were fit with a product of a sinusoid and an exponential decay with a phase offset, $Ae^{-\tau/T_{2E}}\sin(2\pi\Delta\tau + \phi) + C$.

The addition of a TWPA in the second cooldown did not alter any of the sequences. The TWPA pump was pulsed on for the duration of the readout pulse and a 100 ns buffer on each side of the readout pulse.

6. PURCELL LIMIT, DEPHASING, AND CHARGE NOISE

Purcell limit

We estimate the single-mode Purcell rate Γ_P of the qubits to ensure that the device performance is not limited by the Purcell effect. This is done in the non-rotating wave approximation frame using [7],

$$\Gamma_P = 4k_{\text{res}} \left(\frac{\omega_{\text{qubit}}}{\omega_{\text{res}}}\right)^3 \left(\frac{g}{\omega_{\text{res}}}\right)^2, \tag{4}$$

$$T_1^P = \frac{1}{\Gamma_P}, \tag{5}$$

where κ_{res} is the resonator decay rate. The qubit-resonator coupling strength, g is given by,

$$g = \sqrt{\frac{\chi\Delta(\Delta + \alpha)}{\alpha}}, \tag{6}$$

where $\Delta/2\pi = f_{\text{qubit}} - f_{\text{res}}$ is the detuning, χ is the dispersive shift, and α is the anharmonicity. The values of $\kappa_{\text{res}}, \alpha, \chi, \Delta, g$, and T_1^P are reported in Supplementary Table S2. It is evident that even for the qubit with the lowest T_1^P, Q-20 ($T_1^P = 6.1$ ms, $T_1 = 184.8$ ms), the Purcell limit is sufficiently far from the relaxation time.

Thermal photon population and shot noise dephasing

We estimate the thermal photon population for a fully thermalized resonator using the Bose-Einstein distribution [8],

$$n_{BE}(f_{\mathrm{res}}, T) = \frac{1}{e^{hf_{\mathrm{res}}/k_B T} - 1}, \tag{7}$$

where h is the Planck constant, k_B is the Boltzmann constant, and T is the temperature. We take into account thermal photons from higher stages using the equation [8],

$$n_i(f_{\mathrm{res}}) = \frac{n_{i-1}(f_{\mathrm{res}})}{A_i} + \frac{A_i - 1}{A_i} n_{BE}(f_{\mathrm{res}}, T_i), \tag{8}$$

where $A_i > 1$ is the attenuation at a given stage i (20 dB=$10^{20/10} = 100$). Conducting the cascading calculation, we find $\bar{n} \approx 1.9 \times 10^{-2}$ (1.86%) for fridge 1 and $\bar{n} \approx 3.7 \times 10^{-3}$ (0.36%) for fridge 2. With the significant attenuation from the isolator (60 dB), the thermal photons from the output side are negligible in relation to the input.

We estimate bounds on the photon-induced dephasing rate Γ_φ^p of the qubit using the standard formulas [9, 10],

$$\Gamma_\varphi^p = \bar{n}\kappa_{\mathrm{res}} \frac{\chi^2}{\kappa_{\mathrm{res}}^2 + \chi^2}, \tag{9}$$

$$Q_\varphi^p = \frac{2\pi f_{\mathrm{qubit}}}{\Gamma_\varphi^p}, \tag{10}$$

where $\bar{n}$ is the residual photons in the resonator, κ_{res} is the resonator decay rate, χ is the dispersive shift, and f_{qubit} is the qubit frequency. If we assume $\bar{n} \approx 0.02$ (higher of the two calculated thermal photon populations above), we find Q_φ^p to be lowest for Q-7, about 18.7 million ($\mathrm{T}_\varphi^p = 1.1$ ms), and highest for Q-20, at 122 million ($T_\varphi^p = 6.9$ ms). This is consistent with the lack of obvious saturation in Q_φ in the data. Therefore, we infer that the qubits are unlikely to be limited by resonator shot noise dephasing.

Charge noise

We estimate E_J/E_C by using a root-finding algorithm which uses the energy levels calculated via numerically diagonalizing the Hamiltonian [11],

$$\hat{H} = 4E_C(\hat{n} - n_g)^2 - E_J \cos\hat{\phi}, \tag{11}$$

with offset charge $n_g = 0.25$ and the experimentally measured f_{01} and $-\alpha$ to determine the corrected E_J and E_C.

We estimate charge dispersion ϵ_{01} using the same numerical diagonalization process with the corrected E_J and E_C, to solve the equation [11],

$$\epsilon_{01} = E_{01}(n_g = 0.5) - E_{01}(n_g = 0), \tag{12}$$

which yields values in the $10 - 50$ kHz range. Finally, we use ϵ_{01} to calculate the charge noise amplitude A_C using the formula [12],

$$A_C = \frac{1}{2\pi^2\sqrt{\ln 2}} \frac{1}{T_\varphi \epsilon_{01}}. \tag{13}$$

A_C is plotted alongside T_φ as a function of the capacitor gap in Fig. 4(e), with the numbers reported in Table S2. The values of A_C for Q-7, Q-10, Q-20, and Q-150 fall in the range $4 - 8 \times 10^{-3}$ e/$\sqrt{\mathrm{Hz}}$, higher than the generally quoted 1×10^{-3} e/$\sqrt{\mathrm{Hz}}$ [6]. For the calculation conducted here, we assumed that all of the T_φ is attributed to the charge dispersion to examine if the device is charge noise limited primarily due to the E_J/E_C. Therefore, the A_C calculated here can be treated as an upper bound to the $1/f$ charge noise amplitude of the device. If so, then moving to a design with a higher E_J/E_C ratio will alleviate the limitation, and we should observe higher T_φ values in those qubits, irrespective of T_1 and T_{2E} values. We leave a further investigation on the source of dephasing to future work.

A_C for Q-5, however, is significantly larger than any of the other calculated A_C, and higher than A_C measured in significantly lower E_J/E_C qubits [13]. Q-5, having only 5 µm capacitor gaps in an interdigitated design, is significantly

more sensitive to the losses on the surface of the pads (mutual capacitance $C_{12}/C_{\Sigma} \approx 0.85$). The calculated upper bound to the charge noise amplitude is therefore likely well above the actual value.

The trend in the A_C does not seem to fully capture the relative increases in T_{φ}. The increase in E_J/E_C and the capacitor gap would also result in a decreasing sensitivity to TLS-related charge fluctuations within the capacitor pads as well (due to reduced mutual capacitance), which would improve the pure dephasing of the qubits. Thus, it is likely that it is both the charge dispersion, TLS sensitivity, and quasiparticles in combination that results in the T_{φ} trend that we observe here.

Alternatively, if we assume that the charge noise in the qubit is limited to around 1×10^{-3} e/$\sqrt{\mathrm{Hz}}$ for all qubits, which is generally assumed for transmons, then there is a significant additional effect that is limiting the dephasing times in the sample, likely from regions away from the Josephson junction. This could be due to the TLSs present in the capacitor pads or the ground near the qubit.

7. FRIDGE WIRING DIAGRAM

The wiring diagram for the two Bluefors LD250 fridges used for qubit measurement are shown in Fig. S8. Both fridges have a base temperature below 10 mK. Both measurements were conducted with the same Quantum Machines OPX+ and Octave. The addition of room temperature filters and the TWPA line did not significantly affect the measurements in any meaningful way. Any variation, although can be due to variations between fridges, can also be explained by the two week room temperature aging of the qubit.

8. FURTHER FLUCTUATION DATA

Allan deviation and power spectral density (PSD) analysis were conducted on the same time trace data. Overlapping Allan deviation was extracted by using the AllanTools package (v2024.6) [14], with the maximum integration time set to $T_{total}/4$ to ensure statistical significance. The Allan deviation is presented in units of µs to be compared to the T_1 being measured.

PSD analysis was conducted using the Welch method on the time trace with the mean subtracted ($T_1 - \mu_{T_1}$). The spectral density calculation was done with 128 points in each segment and a periodic Hann window. 1/f trend line is

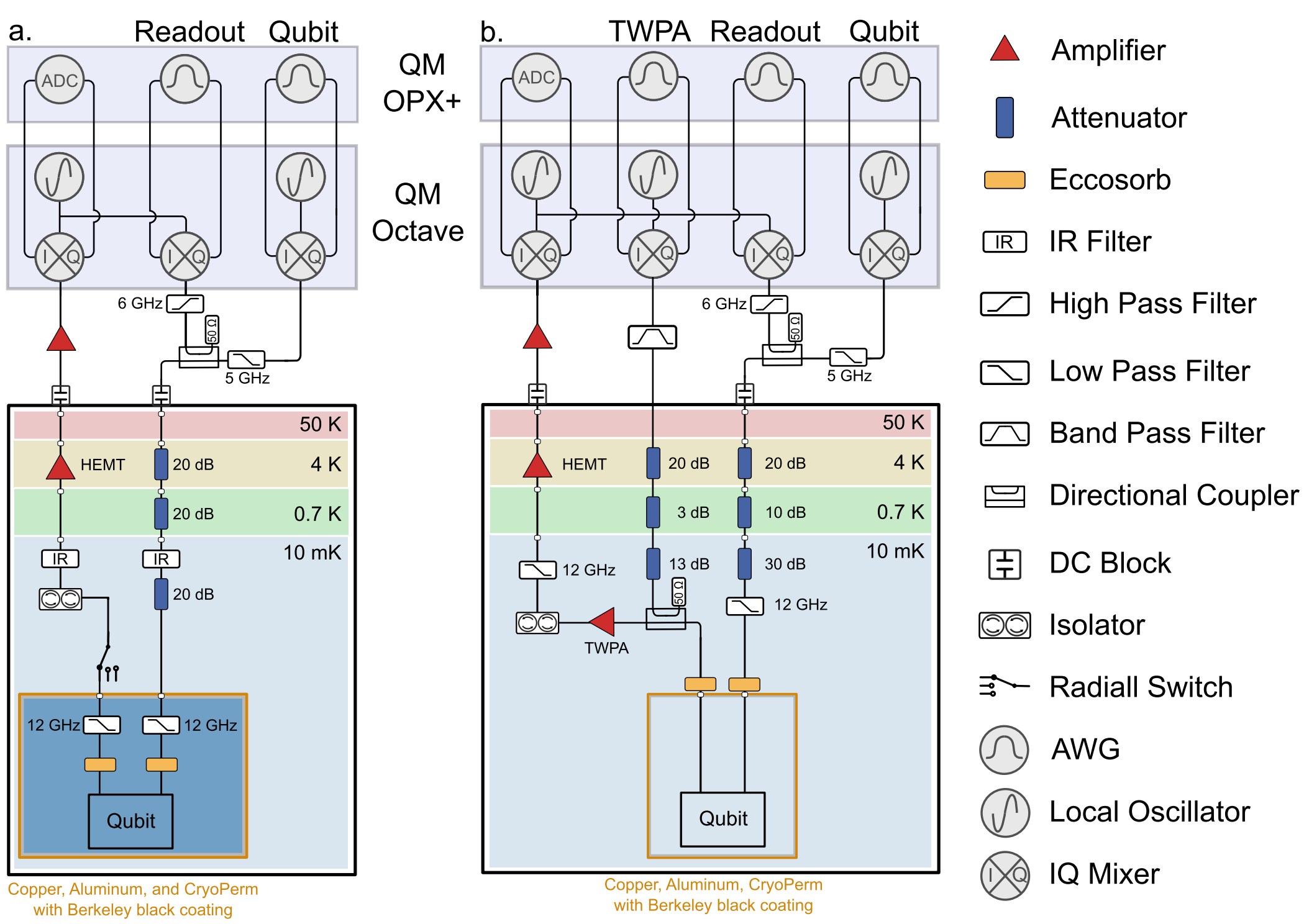

FIG. S8. Fridge wiring diagrams for (a) cooldown 1 in fridge 1, and (b) cooldown 2 in fridge 2.

plotted by using the second datapoint of the PSD as the initialization, followed by a slope of -1. The white noise line in both plots has the same amplitude, defined as $A_W = 6 \times 10^3$ µs^2 Hz^{-1} in the PSD graph, and $\sqrt{A_W/\tau}$ in the Allan deviation plot. Note that neither of these are fitted in the analysis, and present for illustrative purposes as a reference towards understanding the trends observed within the plots.

We conduct the overlapping Allan deviation and PSD statistical analysis on the T_1 fluctuations of all 5 qubits in the second cooldown, hereby referred to as Q-5, Q-7, Q-10, Q-20, and Q-150, denoting their capacitor gap size in microns. The complete dataset is shown in Fig. S9. Note that the time interval between individual measurements is around 36 s for Q-150, while it is around 60 s for all others. Also, there was a 2-hour interruption in data collection for Q-5. Thus, for Allan deviation and PSD only use the latter part of the data and omit the first 1.5 h in the analysis. Also note the difference in the total time of data collection of Q-150 compared to the rest. Data from Q-150 therefore provides better statistics and lower noise compared to the other four.

Q-5, Q-7, and Q-20 have a reduced T_1 compared to the first cooldown, by roughly 10 µs, 15 µs, and 17 µs respectively. We see that all 5 time traces show switching events where the T_1 deviates, usually downwards [Fig. S9(a)-(e)]. Regardless, the T_1 fluctuations remain in a mostly Gaussian distribution [Fig. S9(f)-(j)], implying that any TLS-related fluctuation does not significantly affect the overall shape of the Gaussian. Q-20 is a notable exception, showing a jump in relaxation and coherence near the 15 h mark, creating a clear second distribution at a lower value. This results in a higher peak in the Allan deviation. This implies that although we do not see such jumps in the intervals of measurement of the other qubits, a more in-depth study would be needed to assess the prevalence of dropouts commonly associated with TLSs in these Josephson junctions compared to traditional fabrication methods. The overlapping Allan deviation shows notable differences between the 5 qubits [Fig. S9(k)-(o)]. All qubits have deviations that scale roughly with the T_1, showing similar ratios to the Q-150, indicating that the reduction in T_1 fluctuation is systematic rather than specific to Q-150. The deviation of Q-20, which has the highest deviation of the qubits measured, is still comparable to other qubits [15], highlighting that the overall reduction in fluctuation of the trench-based qubit still holds.

Another clear difference within the Allan deviation plots is the appearance of more significant (or multiple) peaks within the curve, indicative of Lorentzian noise processes. As established in [15, 16], these Lorentzian components originate from telegraph switching due to strongly coupled TLS. This is an indicator of near-resonant TLSs inducing changes in the coherence time on those respective timescales. Q-150 does not contain any significant peak below $\sim 10^4$ s, which as mentioned in the main text, could be due to a near-resonant TLS or some external environmental noise.

Finally, examining the PSD plots for all qubits [Fig. S9(p)-(t)], we see that they are all significantly noisier compared to Q-150 due to the 5-6× reduction in the number of datapoints. They all seem to show 1/f-like behavior initially, followed by a saturation due to the white noise floor. Any Lorentzian process would be difficult to identify within the

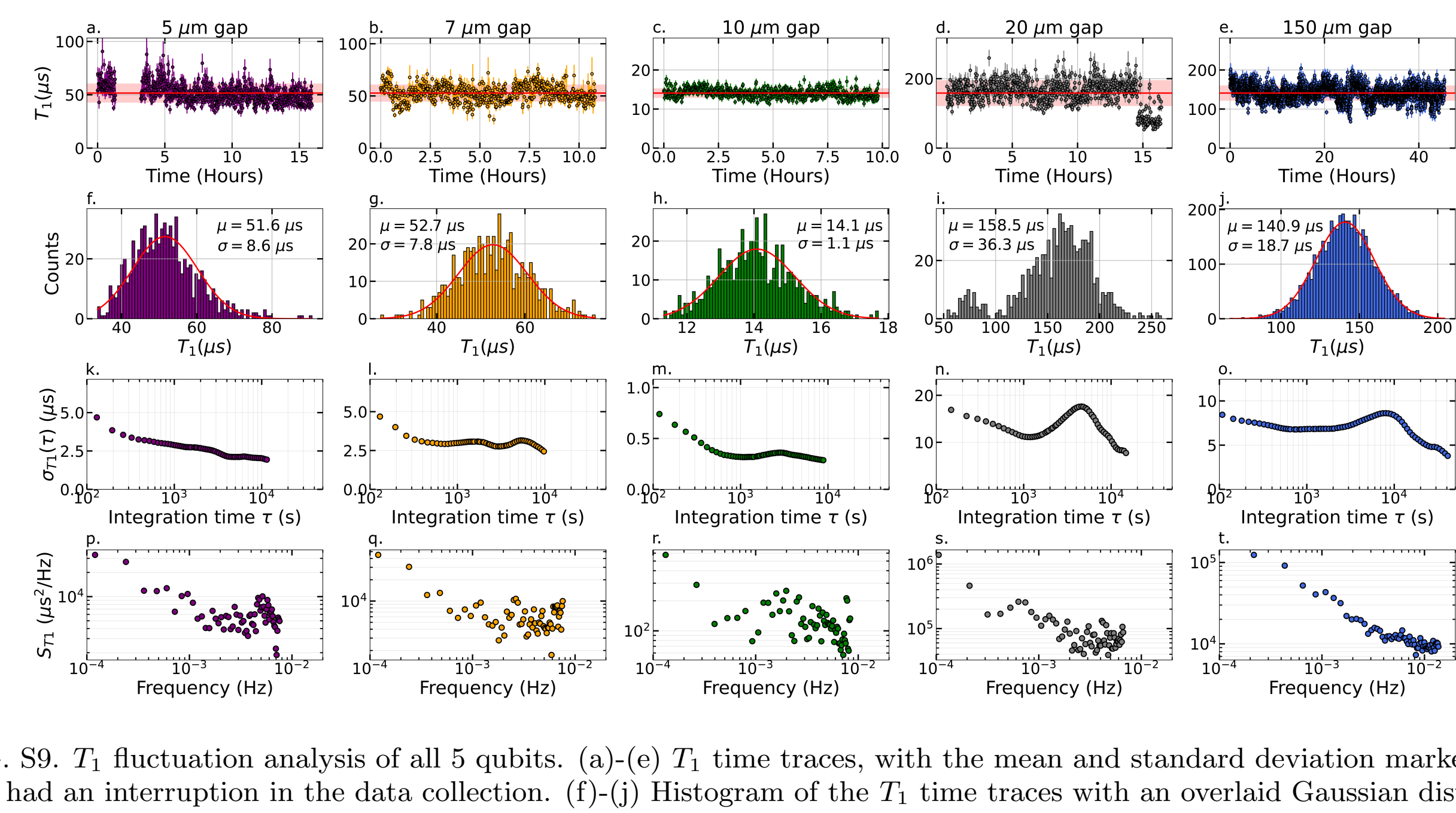

FIG. S9. T_1 fluctuation analysis of all 5 qubits. (a)-(e) T_1 time traces, with the mean and standard deviation marked in red. Q-5 had an interruption in the data collection. (f)-(j) Histogram of the T_1 time traces with an overlaid Gaussian distribution (red) with calculated mean μ and standard deviation σ. (k)-(o) Overlapping Allan deviation with a sliding window of $T_{\text{total}}/4$. Q-5 does not use the initial 1.5 h for the analysis. (p)-(t) Power spectral density calculated using the Welch method with 128 points per segment.

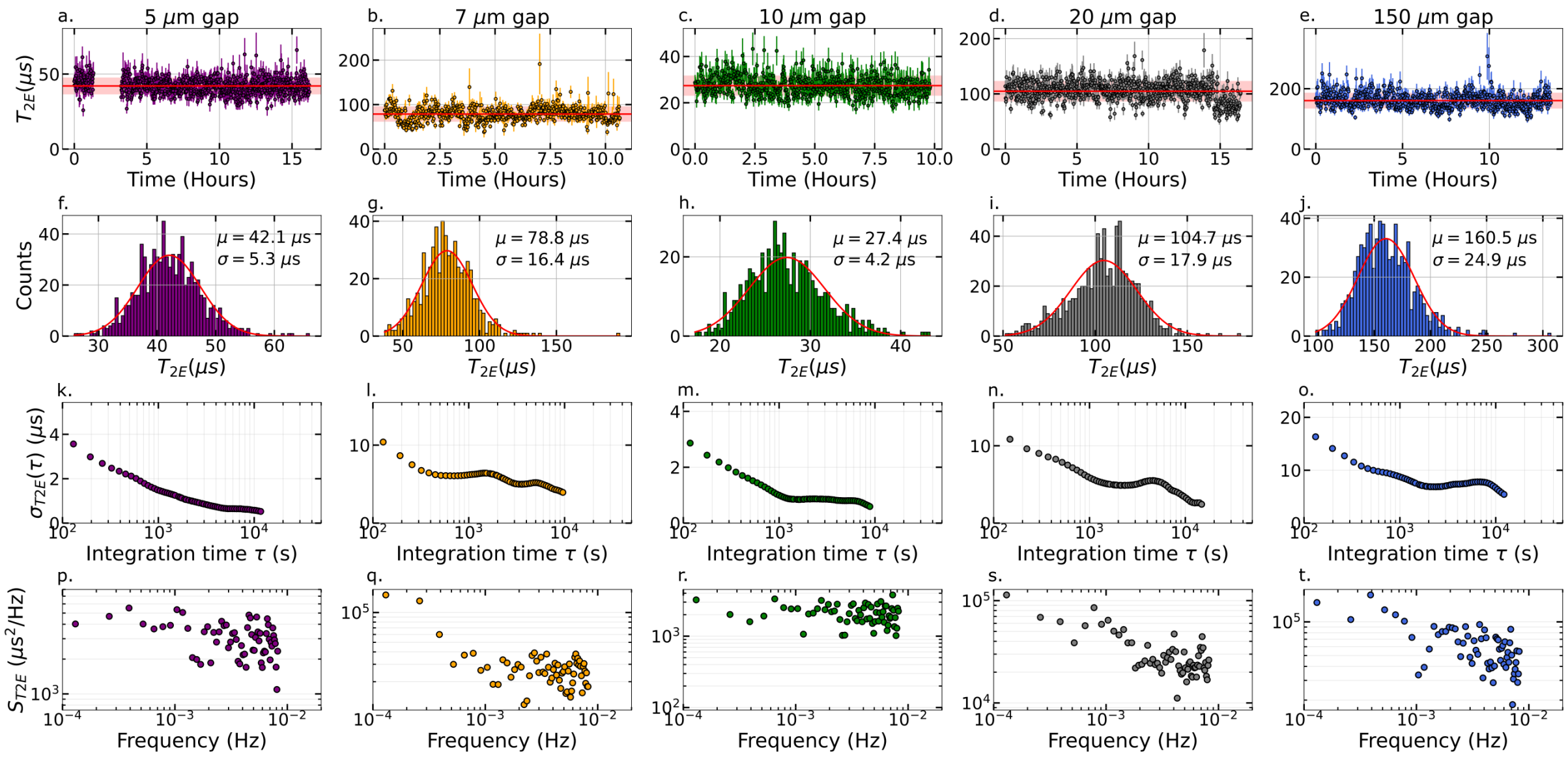

FIG. S10. T_{2E} fluctuation analysis of all 5 qubits. (a)-(e) T_{2E} time traces, with the mean and standard deviation marked in red. Q-5 had an interruption in the data collection. (f)-(j) Histogram of the T_{2E} time traces with an overlaid Gaussian distribution (red) with calculated mean μ and standard deviation σ. (k)-(o) Overlapping Allan deviation with a sliding window of $T_{\text{total}}/4$. Q-5 does not use the initial 1.5 h for the analysis. (p)-(t) Power spectral density calculated using the Welch method with 128 points per segment.

PSD plot. The frequency resolution is around 200 µHz for the PSD plot, which already shows significant noise. It is possible that there could be TLS-associated noise at higher frequency resolution, which would also be hidden by the noise.

We also conduct identical analyses on the T_{2E} data from the 5 qubits, shown in Fig. S10. For Q-5, Q-7, Q-10, and Q-20, the T_{2E} data was collected in an interleaved manner with the T_1 data in Fig. S9. For Q-150, the T_{2E} data was collected at a different time to the data in Fig. S9.

The data shows that the T_{2E} has changed for Q-20 and Q-150 from the first cooldown by a notable amount. The distributions have a larger number of outliers compared to the T_1 fluctuations, resulting in broader tails in all distributions. Not all distributions are symmetric and Gaussian in this instance, with Q-5 being narrower [Fig. S10(f)] and Q-10 having a skew towards higher values [Fig. S10(i)].

It is notable that the Allan deviation is higher for the T_{2E} fluctuations compared to the T_1 fluctuations. They still mimic the features seen in the T_1 plots, such as the two peaks in Q-7 or the peak in Q-150. The PSD data is similarly noisy as in Fig. S9, and the 1/f-like behavior is not seen in Q-5 or Q-10, which seem to be at the white noise floor. This may be due to TWPA pump leakage, which may affect T_{2E} fluctuations. The others show some decay, most notably in Q-7, which matches the T_1 PSD curve quite closely.

9. ROBUST COEFFICIENT OF VARIATION COMPARISON

T_1 time traces are reported on more frequently in place of Allan deviations, alongside standard statistical information from box plots such as medians and interquartile range (IQR). This enables a comparison against other works using the robust coefficient of variation (RCV) [17], defined as the ratio of the IQR (first-third quartile) to the median. This is an incomplete metric for comparison, as these numbers depend significantly on the number of datapoints, sampling rate, and many other factors that affect such measurements. Regardless, this can be used as a simple measure to have some quantitative comparison, while being limited in its ability to directly determine objective superiority. For papers with published box plots, IQR and median were directly extracted from the plots [18–23]. For papers that contain histograms or mean and standard deviation information only, the data was assumed to be Gaussian and symmetric, and the IQR was calculated as $\approx 1.349\sigma$ [16, 24, 25]. We note that this assumption is inaccurate, as the IQR may not be coupled to the σ well due to the high possibility of skewed or non-Gaussianity of the T_1 distributions, in which case the RCV calculation will be different than the true value. Regardless, the works that do contain this information are nominally Gaussian, and we assume that the information is provided with some degree of representativeness of the

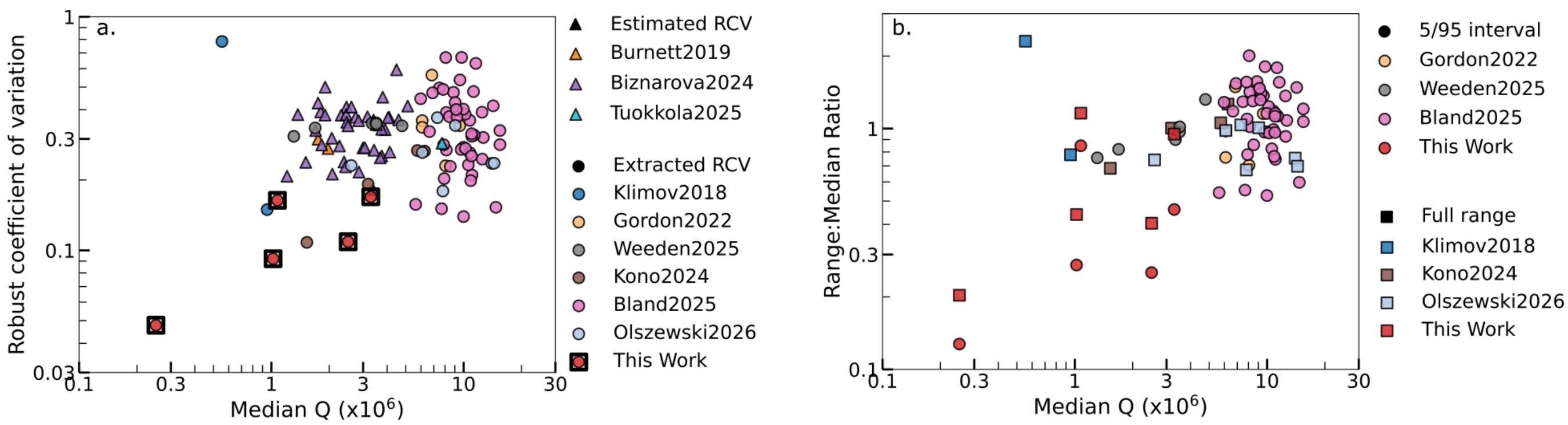

FIG. S11. (a) Robust coefficient of variation (RCV) as a function of quality factor Q for select recent works [16, 18–20, 23–26] in comparison to the work done here [Fig. 4(c)]. RCV was either extracted directly from box plots in the respective works (Extracted RCV, circles) or calculated assuming a Gaussian distribution using the given mean and standard deviation, which provides a reasonable estimation (Estimated RCV, triangles). RCV tends to be at or above 0.2 for most works. The highest RCV from the qubits measured in this work is 0.19. (b) Normalized range as a function of Q. This is plotted for a limited number of datasets which provide the either the full range (squares) or the 5/95 interval (circles) [18–20, 22, 23, 26].

overall distribution. Therefore, the IQR and σ should be correlated to provide a relatively representative RCV.

Fig. S11(a) plots the RCV as a function of quality factor Q for a number of works in the field in recent years [16, 18–20, 22–24, 26], with our data from Fig. 4(c). The RCV tends to have values above 0.2 for most works regardless of the median Q, although they contain outliers that fall below the 0.2 value. The 5 qubits measured here show RCV consistently below 0.2 (highest being 0.19). This suggests that the qubits made using the trench process result in lower fluctuations compared to recent works with comparable quality factors. We also plot the ratio of the range (or 5/95 interval) to the median Q where possible in Fig. S11(b) to illustrate the difference in the full distribution against other works [18–20, 22, 23, 26]. Moving forward, standardization of time-fluctuation measurement and analysis methods will help to improve comparability.

[1] V. Ambegaokar and A. Baratoff, Tunneling Between Superconductors, Physical Review Letters **10**, 486 (1963).
[2] C. Križan, M. Toselli, I. Ahmad, H. Khaksaran, M. Rommel, N. Trnjanin, J. Biznárová, M. Dahiya, E. Hogedal, H. Jakobsson, A. Nylander, J. Bylander, P. Delsing, and G. Tancredi, Electrical post-fabrication tuning of aluminum Josephson junctions at room temperature, Materials for Quantum Technology **6**, 036001 (2026).
[3] S. Lazar, P. Tiemeijer, C. S. Schnohr, M. Meledina, C. Patzig, T. Höche, P. Longo, and B. Freitag, Enabling electron-energy-loss spectroscopy at very high energy losses: An opportunity to obtain x-ray absorption spectroscopy–like information using an electron microscope, Physical Review Applied **23**, 054095 (2025).
[4] S. D. Funni and C. Evans, Sdfunni/SingleOrigin: SingleOrigin v3.0b2, Zenodo (2025).
[5] C. J. Kopas, E. Lachman, C. R. H. McRae, Y. Mohan, J. Y. Mutus, A. Nersisyan, and A. Poudel, Simple coplanar waveguide resonator mask targeting metal-substrate interface (2022), accessed: June 3, 2026, arXiv:2204.07202 [quant-ph].
[6] P. Krantz, M. Kjaergaard, F. Yan, T. P. Orlando, S. Gustavsson, and W. D. Oliver, A quantum engineer's guide to superconducting qubits, Applied Physics Reviews **6**, 021318 (2019).
[7] P. D. Kurilovich, T. Connolly, C. G. L. Bøttcher, D. K. Weiss, S. Hazra, V. R. Joshi, A. Z. Ding, H. Nho, S. Diamond, V. D. Kurilovich, W. Dai, V. Fatemi, L. Frunzio, L. I. Glazman, and M. H. Devoret, High-frequency readout free from transmon multi-excitation resonances (2025), accessed: July 30, 2026, arXiv:2501.09161 [quant-ph].
[8] S. Krinner, S. Storz, P. Kurpiers, P. Magnard, J. Heinsoo, R. Keller, J. Luetolf, C. Eichler, and A. Wallraff, Engineering cryogenic setups for 100-qubit scale superconducting circuit systems, EPJ Quantum Technology **6**, 2 (2019).
[9] A. P. Sears, A. Petrenko, G. Catelani, L. Sun, H. Paik, G. Kirchmair, L. Frunzio, L. I. Glazman, S. M. Girvin, and R. J. Schoelkopf, Photon shot noise dephasing in the strong-dispersive limit of circuit QED, Physical Review B **86**, 180504 (2012).
[10] F. Yan, S. Gustavsson, A. Kamal, J. Birenbaum, A. P. Sears, D. Hover, T. J. Gudmundsen, D. Rosenberg, G. Samach, S. Weber, J. L. Yoder, T. P. Orlando, J. Clarke, A. J. Kerman, and W. D. Oliver, The flux qubit revisited to enhance coherence and reproducibility, Nature Communications **7**, 12964 (2016).
[11] J. Koch, T. M. Yu, J. Gambetta, A. A. Houck, D. I. Schuster, J. Majer, A. Blais, M. H. Devoret, S. M. Girvin, and R. J. Schoelkopf, Charge-insensitive qubit design derived from the Cooper pair box, Physical Review A **76**, 042319 (2007).
[12] G. Ithier, E. Collin, P. Joyez, P. J. Meeson, D. Vion, D. Esteve, F. Chiarello, A. Shnirman, Y. Makhlin, J. Schriefl, and G. Schön, Decoherence in a superconducting quantum bit circuit, Physical Review B **72**, 134519 (2005).
[13] B. G. Christensen, C. D. Wilen, A. Opremcak, J. Nelson, F. Schlenker, C. H. Zimonick, L. Faoro, L. B. Ioffe, Y. J. Rosen, J. L. DuBois, B. L. T. Plourde, and R. McDermott, Anomalous charge noise in superconducting qubits, Physical Review B **100**, 140503 (2019).
[14] A. Wallin, Aewallin/allantools (2026).
[15] F. Berritta, J. Benestad, J. A. Krzywda, O. Krause, M. A. Marciniak, S. Krøjer, C. W. Warren, E. Hogedal, A. Nylander, I. Ahmad, A. Osman, J. Biznárová, M. Rommel, A. F. Roudsari, J. Bylander, G. Tancredi, J. Danon, J. Hastrup, F. Kuemmeth, and M. Kjaergaard, Real-Time Adaptive Tracking of Fluctuating Relaxation Rates in Superconducting Qubits, Physical Review X **16**, 011025 (2026).
[16] J. J. Burnett, A. Bengtsson, M. Scigliuzzo, D. Niepce, M. Kudra, P. Delsing, and J. Bylander, Decoherence benchmarking of superconducting qubits, npj Quantum Information **5**, 54 (2019).
[17] Z. Botta-Dukát, Quartile coefficient of variation is more robust than CV for traits calculated as a ratio, Scientific Reports **13**, 4671 (2023).
[18] M. W. Olszewski, L. Kong, S. Reinhardt, D. Tong, X. Du, G. Di Gianluca, H. Lu, S. Roy, L. Zhang, A. B. Biedron, D. A. Muller, and V. Fatemi, Krypton-sputtered tantalum films for scalable high-performance quantum devices, Nature Materials 10.1038/s41563-026-02718-z (2026).
[19] R. T. Gordon, C. E. Murray, C. Kurter, M. Sandberg, S. A. Hall, K. Balakrishnan, R. Shelby, B. Wacaser, A. A. Stabile, J. W. Sleight, M. Brink, M. B. Rothwell, K. P. Rodbell, O. Dial, and M. Steffen, Environmental radiation impact on lifetimes and quasiparticle tunneling rates of fixed-frequency transmon qubits, Applied Physics Letters **120**, 074002 (2022).
[20] S. Kono, J. Pan, M. Chegnizadeh, X. Wang, A. Youssefi, M. Scigliuzzo, and T. J. Kippenberg, Mechanically induced correlated errors on superconducting qubits with relaxation times exceeding 0.4 ms, Nature Communications **15**, 3950 (2024).
[21] D. Colao Zanuz, Q. Ficheux, L. Michaud, A. Orekhov, K. Hanke, A. Flasby, M. Bahrami Panah, G. J. Norris, M. Kerschbaum, A. Remm, F. Swiadek, C. Hellings, S. Lazăr, C. Scarato, N. Lacroix, S. Krinner, C. Eichler, A. Wallraff, and J.-C. Besse, Mitigating losses of superconducting qubits strongly coupled to defect modes, Physical Review Applied **23**, 044054 (2025).
[22] M. P. Bland, F. Bahrami, J. G. C. Martinez, P. H. Prestegaard, B. M. Smitham, A. Joshi, E. Hedrick, S. Kumar, A. Yang, A. C. Pakpour-Tabrizi, A. Jindal, R. D. Chang, G. Cheng, N. Yao, R. J. Cava, N. P. De Leon, and A. A. Houck, Millisecond lifetimes and coherence times in 2D transmon qubits, Nature **647**, 343 (2025).
[23] P. V. Klimov, J. Kelly, Z. Chen, M. Neeley, A. Megrant, B. Burkett, R. Barends, K. Arya, B. Chiaro, Y. Chen, A. Dunsworth, A. Fowler, B. Foxen, C. Gidney, M. Giustina, R. Graff, T. Huang, E. Jeffrey, E. Lucero, J. Y. Mutus, O. Naaman, C. Neill, C. Quintana, P. Roushan, D. Sank, A. Vainsencher, J. Wenner, T. C. White, S. Boixo, R. Babbush, V. N. Smelyanskiy, H. Neven, and J. M. Martinis, Fluctuations of Energy-Relaxation Times in Superconducting Qubits, Physical Review Letters **121**, 090502 (2018).
[24] J. Biznárová, A. Osman, E. Rehnman, L. Chayanun, C. Križan, P. Malmberg, M. Rommel, C. Warren, P. Delsing, A. Yurgens, J. Bylander, and A. Fadavi Roudsari, Mitigation of interfacial dielectric loss in aluminum-on-silicon superconducting qubits, npj Quantum Information **10**, 78 (2024).

[25] M. Tuokkola, Y. Sunada, H. Kivijärvi, J. Albanese, L. Grönberg, J.-P. Kaikkonen, V. Vesterinen, J. Govenius, and M. Möttönen, Methods to achieve near-millisecond energy relaxation and dephasing times for a superconducting transmon qubit, Nature Communications **16**, 5421 (2025).

[26] S. Weeden, D. Harrison, S. Patel, M. Snyder, E. Blackwell, G. Spahn, S. Abdullah, Y. Takeda, B. Plourde, J. Martinis, and R. McDermott, Statistics of strongly coupled defects in superconducting qubits, Physical Review Applied **25**, 044050 (2026).
